# M2 macrophage-derived TGF-β induces age-associated loss of adipogenesis through progenitor cell senescence

**DOI:** 10.1016/j.molmet.2024.101943

**Published:** 2024-04-23

**Authors:** Xinyi Zeng, Teh-Wei Wang, Kiyoshi Yamaguchi, Seira Hatakeyama, Satoshi Yamazaki, Eigo Shimizu, Seiya Imoto, Yoichi Furukawa, Yoshikazu Johmura, Makoto Nakanishi

**Affiliations:** 1 Division of Cancer Cell Biology, The Institute of Medical Science, The University of Tokyo, 4-6-1 Shirokanedai, Minato-ku, Tokyo 108-8639, Japan; 2 Division of Clinical Genome Research, The Institute of Medical Science, The University of Tokyo, 4-6-1 Shirokanedai, Minato-ku, Tokyo 108-8639, Japan; 3 Division of Stem Cell Biology, The Institute of Medical Science, The University of Tokyo, 4-6-1 Shirokanedai, Minato-ku, Tokyo 108-8639, Japan; 4 Division of Health Medical Intelligence, Human Genome Center, The Institute of Medical Science, The University of Tokyo, 4-6-1 Shirokanedai, Minato-ku, Tokyo 108-8639, Japan; 5 Division of Cancer and Senescence Biology, Cancer Research Institute, Institute for Frontier Science Initiative, Kanazawa University, Kanazawa, Japan

**Keywords:** Adipogenesis, Senescence, p16, Adipose progenitor cells, TGF-β, Cachexia

## Abstract

**Objectives:**

Adipose tissue is an endocrine and energy storage organ composed of several different cell types, including mature adipocytes, stromal cells, endothelial cells, and a variety of immune cells. Adipose tissue aging contributes to the pathogenesis of metabolic dysfunction and is likely induced by crosstalk between adipose progenitor cells (APCs) and immune cells, but the underlying molecular mechanisms remain largely unknown. In this study, we revealed the biological role of p16^high^ senescent APCs, and investigated the crosstalk between each cell type in the aged white adipose tissue.

**Methods:**

We performed the single-cell RNA sequencing (scRNA-seq) analysis on the p16^high^ adipose cells sorted from aged p16-Cre^ERT2^/Rosa26-LSL-tdTomato mice. We also performed the time serial analysis on the age-dependent bulk RNA-seq datasets of human and mouse white adipose tissues to infer the transcriptome alteration of adipogenic potential within aging.

**Results:**

We show that M2 macrophage-derived TGF-β induces APCs senescence which impairs adipogenesis *in vivo*. p16^high^ senescent APCs increase with age and show loss of adipogenic potential. The ligand–receptor interaction analysis reveals that M2 macrophages are the donors for TGF-β and the senescent APCs are the recipients. Indeed, treatment of APCs with TGF-β1 induces senescent phenotypes through mitochondrial ROS-mediated DNA damage *in vitro*. TGF-β1 injection into gonadal white adipose tissue (gWAT) suppresses adipogenic potential and induces fibrotic genes as well as p16 in APCs. A gWAT atrophy is observed in cancer cachexia by APCs senescence, whose induction appeared to be independent of TGF-β induction.

**Conclusions:**

Our results suggest that M2 macrophage-derived TGF-β induces age-related lipodystrophy by APCs senescence. The TGF-β treatment induced DNA damage, mitochondrial ROS, and finally cellular senescence in APCs.

## Highlights

• The p16^high^ APCs show the lower adipogenic potential.
• Cellular senescence of APCs is induced by TGF-β secreted by M2 macrophages.
• Time serial analysis reveal the transcriptomic changes in macrophages in WAT during aging.
• Increase in the p16^high^ APCs is involved in cancer cachexia.

## 1 Introduction

Cellular senescence refers to the state in which cells enter permanent cell cycle arrest in response to environmental cellular stresses. Besides, senescent cells are characterized by the production of numerous pro-inflammatory cytokines, growth factors, and other secreted factors, collectively known as the senescence-associated secretory phenotype (SASP) [1]. These senescent cells progressively accumulate during the aging process of an organism, leading to localized chronic inflammation in tissues.

Adipose tissue plays a critical role in maintaining systemic glucose, lipid, and energy balance. It is also involved in the secretion of several adipokines and endocrine hormones [2]. During the aging process, adipose tissue gradually loses its ability to maintain the balance of homeostasis. In general, white adipose tissue (WAT) exhibits characteristic signs of aging, including an increase in adipocyte size (hypertrophy) and a reduced capacity for adipogenesis [3]. As a result, WAT becomes inefficient in storing excess energy in the body, leading to the development of various metabolic disorders [4]. At the same time, aging adipose tissue exhibits chronic inflammation by upregulating the expression of pro-inflammatory cytokines, which further contributes to the increased risk of cardiovascular diseases and type 2 diabetes [5].

Over the past decade, numerous studies have suggested that many phenotypic manifestations of adipose tissue aging may be associated with the accumulation of senescent cells. In aged WAT, the expression levels of the senescence marker genes, p21 and p16, increase with age [6,7]. Furthermore, the association between the accumulation of senescent cells and adipose tissue fibrosis or insulin resistance has been previously described [8,9]. SASP factors promote adipose tissue fibrosis, leading to structural changes and impaired metabolic functions. In addition, the presence of senescent cells in adipose tissue contributes to dysregulating insulin signaling pathways and the development of insulin resistance in aging. Studies using genetic and pharmacological approaches to eliminate senescent cells in WAT have shown promising results in terms of improving insulin sensitivity and reducing adipose tissue inflammation [6,10,11]. Recently, adipocytes have become highly susceptible to senescence in obesity and activation of sterol regulatory element-binding protein 1c (SREBP1c)-PARP1 axis counteracts the senescence program, the loss of which exacerbates systemic insulin resistance [12]. In the culture system, senescent APCs secrete activin A, which suppresses the adipogenesis of surrounding APCs [13,14]. These findings underscore the critical role of senescent cells in adipose tissue dysfunction and its implications for age-related metabolic disorders. However, much remains unknown about the specific cell types involved in cellular senescence and how senescent cells participate in WAT aging phenotypes *in vivo*.

Mammalian WAT depots contain diverse populations of immune cells. Among these, adipose tissue macrophages (ATMs) play a central role in the inflammation induced by obesity and aging [3,15]. ATMs are known to exhibit functional heterogeneity. M1 ATMs produce pro-inflammatory cytokines and M2 ATMs have anti-inflammatory potential. The number and ratio of M1 and M2 ATMs affect the adipose tissue microenvironment and regulate insulin sensitivity. The interactions between senescent adipose-derived cells, vascular endothelial cells, and immunoregulatory cells including ATMs within the adipose tissue remain largely unexplored.

In this study, we used p16-Cre^ERT2^/LSL-tdTomato mice to visualize and isolate the adipose-derived cells from aged mice for single-cell RNA sequencing (scRNA-seq) [16]. Using this approach, we analyzed the transcriptomic differences between senescent and non-senescent adipose progenitor cells. In addition, we employ single-cell analysis to investigate the interactions between senescent cells and other vascular endothelial or immune cells within the adipose tissue microenvironment.

## 2 Materials and methods

### 2.1 Mouse models

All animal protocols were approved by the University of Tokyo and the Institutional Laboratory Animal Care, and all experiments were performed according to their guidelines. The mice were housed in a pathogen-free facility, residing in ventilated cages and maintained under controlled conditions (12-h light/dark cycle, 23–25 °C). They had unrestricted access to standard mouse diets (CA-1, CLEA Japan) and water. The p16-Cre^ERT2^-tdTomato mice were generated through the crossing of p16^Ink4a^-Cre^ERT2^ mice [16] with Rosa26-CAG-lsl-tdTomato mice obtained from Jackson's Laboratory. For all experimental groups, age-matched mice of the same strain were used. All the mice utilized in the study were the result of in-house breeding.

### 2.2 Mouse experiments

The male mice were exclusively selected for all experimental procedures. In the case of both young and aging mouse models, p16-Cre^ERT2^-tdTomato mice were employed. These mice were sacrificed at the predetermined time point, precisely two weeks after receiving daily intraperitoneal (i.p.) injections of tamoxifen (TAM) (Sigma–Aldrich) at a dosage of 80 mg/kg body weight for five consecutive days.

To establish cancer cachexia models, we employed 2–3-month-old p16-Cre^ERT2^-tdTomato mice. The model was constructed by subcutaneously inoculating KPCY primary murine pancreatic adenocarcinoma cells kindly provided by Dr. Ben Z. Stanger [17] at an amount of 3 × 10^5^ cells per mouse in 100 μl of PBS, specifically targeting the dorsal region. Five weeks after the tumor cell inoculation, the mice were sacrificed for subsequent analysis. In the last two weeks before sacrifice, TAM was administered via i.p. injections every other day, resulting in a total of seven injections. The body weight and gonadal adipose tissue were monitored and recorded. SB431542 (Selleck) was dissolved in DMSO in 50 mg/ml to prepare the stock solution. In the last two weeks before sacrifice, SB431542 was diluted in corn oil (Wako) and administered via i.p. injections with a dose of 10 mg/kgBW every other day, resulting in a total of seven injections.

For the high-fat diet (HFD) model, we employed 2–3-month-old p16-Cre^ERT2^-tdTomato mice and fed them with an HFD containing 60 kcal% fat, 20 kcal% carbohydrate, and 20 kcal% protein (Research Diets) for either 4 weeks or 8 weeks. Following the dietary intervention, the mice were administered i.p. injections of TAM at a dosage of 80 mg/kg body weight for five consecutive days at the designated time points. Two weeks after the final TAM injection, the mice were sacrificed for subsequent analyses.

For *in vivo* TGF-β1 injection, we followed the in situ injection protocol for AAV delivery [18]. TGF-β1 was reconstituted with PBS to a working solution of 400 μg/ml. Under anesthesia with 3.5% isoflurane, the peritoneal cavity of the mice was opened, and then 8 μl∗6 spots of the solution were injected into one side of the fat pad. A total of 40 μg of TGF-β1 was directly injected into the gWAT of the mice. The mice were sacrificed two weeks after the injection.

### 2.3 RNA extraction and quantitative real-time PCR analysis

Total RNA from sorted cells or cultured cells was extracted using the Single Cell RNA Purification Kit (NORGEN) according to the manufacturer's instructions, and then reverse transcribed into complementary DNA (cDNA) with the ReverTra Ace qPCR RT kit (TOYOBO). Quantitative real-time PCR (qPCR) was carried out on optical 96-well reaction plates (Applied Biosystems) using the THUNDERBIRD® SYBR® qPCR Mix (TOYOBO). The qPCR protocol consisted of an initial pre-incubation step at 95 °C for 1 min, followed by 40 cycles of amplification at 95 °C for 15 s and 60 °C for 30 s. The expression levels of each gene were normalized with Actb. Primer sequences for the target genes were listed in Supplementary Table 1.

### 2.4 Western blotting

Protein extraction from cells was carried out through homogenization in Laemmli buffer containing 2% SDS, 10% glycerol, 5% 2-mercaptoethanol, 0.002% bromophenol blue, and 62.5 mM Tris HCl at pH 6.8. Following extraction, the whole cell lysates (20–50 μg) were separated using SDS-PAGE, transferred onto a PVDF membrane (Immobilon-P; Millipore), and subjected to a standard western blot procedure. The antibodies used in this study are listed in Supplementary Table 2. The images were visualized by using the Amersham Imager 680 (Cytiva).

### 2.5 Isolation of gonadal adipose stromal vascular fraction and flow cytometry analysis

Gonadal adipose tissue samples were subjected to direct mincing using a sharp scissor, followed by digestion in DMEM supplemented with 0.3 unit/ml Liberase TM (Roche) and 10% BSA at 37 °C for 50 min with gentle shaking. After incubation, the resulting cell suspension was centrifuged at 400*g* for 3 min, and the supernatant was removed. The stromal vascular fractions (SVFs) present in the cell pellet were resuspended in DMEM and passed through 100 μm cell strainers. Subsequently, the red blood cells were lysed using RBC buffer for 5 min.

The remaining cells were washed with PBS and resuspended in PBS containing 4% FBS and mouse FcR blocking reagent (MACS) at 4 °C for 10 min, and further stained with desired antibodies at 4 °C for 30 min, washed and subjected to FACS SORP Aria (BD Biosciences). The antibodies used in this study are listed in Supplementary Table 2. All the flow cytometry data were analyzed and graphed using FlowJoV10.7.1.

### 2.6 Single-cell RNA sequencing analysis

For the scRNA-seq library construction, we follow the manufacturer's instructions of 10× Genomics Chromium Single Cell 3′ Reagent Kit v3.1. The single-cell suspension (either whole population or adipocyte progenitor cells) was isolated from the gWAT of 18-month-old p16-Cre^ERT2^-tdTomato mice. Library sequencing was performed on the DNBSEQ-G400RS (MGI Tech) with 150 bp paired-end reads. The Cell Ranger package (version 3.0.2) was utilized to process unique molecular identifiers (UMIs) and barcodes and align the transcripts to a mm10 mouse reference genome. After obtaining the feature-barcode matrix, quality control, clustering, cell annotation, and identification of differentially expressed genes (DEGs) were performed using the R (version 4.2.1) package Seurat (version 4.3.0) [19]. The thresholds of quality control included 1000 < nFeatures < 7000 and mitochondrial counts ratio <8%. After quality control filtering, a total of 3031 Tom^+^ APCs and 4493 Tom^−^ APCs were selected for further normalization, log-transformation, dimensional reduction, and clustering. Marker genes were generated by the FindAllMarkers function using the Wilcoxon Rank Sum test, and then cell clusters were assigned to specific cell populations based on the expression of canonical markers of these cell populations.

The differentially expressed genes (DEGs) between Tom^+^ and Tom^−^ APCs were conducted using the ‘FindMarkers’ function in the Seurat software. The non-parametric two-sided Wilcoxon rank-sum test was employed to calculate the log2 fold changes (Log2FC) and adjusted p-values for each identified DEG calculated by B–H method. Specifically, DEGs with an absolute value of ‘avg_logFC’ greater than 0.1 and a ‘p_val_adj’ less than 0.05 were designated as Tom^+^/Tom^−^ DEGs of APCs.

Gene Ontology (GO) analysis was carried out using Metascape (version 3.5) [20] to identify enriched biological processes, molecular functions, and cellular components associated with the differentially expressed genes (DEGs). The results were visualized using the ggplot2 R package. Representative terms chosen from the top 100 ranked GO terms or pathways with a significance threshold of p-value <0.01 were displayed.

The Gene Set Variation Analysis (GSVA) [21] was conducted using the GSVA R package (version 1.44.5). The signaling pathway gene sets of interest were obtained from the MsigDB database. To facilitate the analysis, the gene-by-cell matrix was converted to a geneset-by-cell matrix, and GSVA scores were computed for gene sets with a minimum of 5 detected genes. Subsequently, the significantly enriched pathways were identified using the limma R package (version 3.52.4) [22]. Only pathways that demonstrated statistical significance in two-sided unpaired t-tests followed by Benjamini–Hochberg p-value adjustment were included for downstream analysis.

The transcriptional regulatory network was examined using the SCENIC (version 1.3.1) workflow [23], specifically employing the GENIE3 (version 1.18.0) [24] and RcisTarget (version 1.16.0) R packages, with default parameters. The reference transcription factors (TFs) for the mm10 genome were obtained using RcisTarget. To begin, co-expression modules were identified by analyzing the gene expression matrix, focusing on the relationship between TFs and potential target genes using GENIE3. Subsequently, for each co-expression module, a cis-regulatory motif enrichment analysis was performed among all potential target genes using RcisTarget. Only target genes showing enrichment for motifs corresponding to the respective TFs were considered direct target genes. Finally, the gene regulatory networks of Tom^+^ and Tom^−^ APCs were inferred.

To investigate the presence of cell–cell communication molecules within Tom^+^ APCs, Tom^−^ APCs, and other cell types, we utilized CellPhoneDB R packages (version 4.0.0) [25,26] to infer the intercellular communication network using the default setting based on single-cell transcriptome data. The visualization of these interactions was accomplished using the netVisual_bubble function. Then, differentially expressed signaling pathways were found by function *rankNet*, and the expression distribution of signaling genes associated with TGF-β signaling pathways across different datasets was visualized by function *plotGeneExpression*. Lastly, the most significant signals contributing to outgoing or incoming signaling within each cell group were identified by function netAnalysis_signalingRole_heatmap function.

### 2.7 RNA-seq analysis of APCs in cancer cachexia model

The total RNA of sorted Tom^+^ and Tom^−^ APCs from tumor-bearing mice was extracted by using Single Cell RNA Purification Kit (NORGEN) according to the manufacturer's instructions. Total RNA was submitted to Novogene (China) and reverse transcribed into the cDNA library. Then the cDNA samples were fragmented, end-repaired, A-tailed, and ligated with adaptors. After size selection and PCR enrichment, the RNA library was sequenced on Illumina platforms.

The sequencing data were aligned to a mouse reference genome (mm10) using Rsubread (version 2.4.3) [27]. Raw counts were obtained from read alignments through refGene, and then further transferred into CPM by edgeR (version 3.32.1) [28]. After filtering out low-expression genes with CPM lower than 10. Differential expression was analyzed with the linear model using limma (version 3.46.0) [22]. Genes with log2FC > 1 and FDR < 0.05 adjusted by B–H method were considered as significant differentially expressed genes (DEGs). For gene set enrichment analysis (GSEA), the order ranked gene list by log2 fold change was inputted into the pre-ranked GSEA function in clusterProfiler (version 3.18.1) [29], and mouse gene sets were obtained from msigdb (v7.4.1).

### 2.8 RNA-seq analysis of published datasets

To investigate the changes in visceral adipose tissue gene expression across the life span of mice, bulk RNA-seq datasets (Tabula Muris Senis project) [30] were obtained from the Gene Expression Omnibus (GEO) database, specifically the accession number GSE132042. A total of 40 visceral adipose tissue samples collected from different ages were included in the analysis. Normalization of gene expression counts was performed using the VST feature within DESeq2 (version 1.36.0) [31] implemented in R (version 4.2.1). Subsequently, the mean gene expression was calculated for each age group, consisting of four samples. From these, the top 3000 variable genes were selected for downstream analysis, which encompassed time-series analysis, Gene Ontology (GO) enrichment analysis, and Fisher's exact test. The time-series genes were subjected to clustering using the Mfuzz R package [32]. Mfuzz utilizes the fuzzy c-means algorithm for soft clustering, employing the average TPM (transcripts per million) values of individual genes as input. For the clustering analysis, the number of clusters was set to 6, and the fuzzifier coefficient (M) was set to 1.5.

To analyze bulk RNA-seq datasets of human tissues, we obtained the raw count RNA-seq datasets from the GTEx (v8) database [33,34]. A total of 371 visceral adipose tissue samples from non-diseased males aged 20–80 years old. The analysis pipeline utilized for processing the bulk RNA-seq data was similar to the approach employed in the Tabula Muris Senis project. First, genes with a read count of 0 were excluded from the samples. Subsequently, the read counts were normalized using the VST feature within DESeq2. Given that the age information of the GTEx donors was recorded in ten-year ranges, the mean gene expression within each age range was calculated. Subsequently, the top 3000 variable genes were selected for further analysis. This included downstream analysis such as time-series analysis, Gene Ontology (GO) enrichment analysis, and Fisher's exact test.

### 2.9 Cell culture

The primary APCs were obtained from the gWAT of male mice via FACS following the protocol outlined in the Flow cytometry section. For the adipogenesis induction, the APCs were directly plated in 24-well plates. The cells were stimulated with adipogenic induction media, consisting of growth media supplemented with 1 μg/ml insulin, 1 μM dexamethasone, and 0.5 mM isobutyl methylxanthine, for a duration of 48 h in a 5% CO_2_ incubator at 37 °C. After that, the media were replaced with growth media containing 1 μg/ml insulin to the growth media, and the media was changed every 2 days for a period of 6–8 days to monitor adipocyte differentiation.

For cytokine stimulation experiments, the APCs were treated with TGF-β1 at a concentration of 10 ng/ml, or with PBS as a control, for a duration of 4 days. In the case of mTOR inhibition experiments, the APCs were pre-treated with rapamycin at a concentration of 0.1 nM for 30 min prior to the stimulation together with TGF-β1 at 10 ng/ml for 4 days.

For the bone marrow-derived macrophages (BMDMs), after removing the femur from the mouse's lower limbs, a 23G needle was inserted into the end of the femur for flushing out bone marrow cells with 10 ml cold PBS. Red blood cells were lysed by RBS lysis buffer (Thermo Fisher). Cells were seeded in non-treated dishes (falcon) and cultured with DMEM + 10% FBS + 1% P/S + 10% CMG 14-12 culture supernatant for 6 days to differentiate into macrophages. The M2 polarization was induced by 20 ng/ml IL-4 (peprotech) for 4 days.

### 2.10 Oil red O staining

The adipocyte progenitor cells (APCs) were subjected to fixation by immersing them in 4% paraformaldehyde (PFA) for 30 min at room temperature. Following the fixation step, the cells were carefully rinsed with distilled water, and then briefly exposed to 60% isopropanol for 5 min. The isopropanol was removed before the application of the Oil red O working solution for 1 h, which comprised a concentration of 1.8 mg/ml Oil red O dissolved in 60% isopropanol. After completion of the incubation, the Oil red O solution was discarded, and the cells were subjected to three consecutive washes with distilled water to ensure the removal of any residual background staining. As a counterstain, a blue hematoxylin solution was added to the APCs for 1 min. Following a final wash with distilled water, the cellular images were acquired for subsequent analysis purposes.

### 2.11 ATP content measurements

The sorted APCs were seeded in 96-well plates at 5 × 10^4^ cells per well in 200 μl of growth media. Following overnight incubation, the cells were exposed to a concentration of 10 ng/ml of TGF-β1, along with fresh growth medium, for a duration of 72 h. To initiate the assays, the growth medium was carefully removed from each well and replaced with 100 μl of PBS. Subsequently, 50 μl of mammalian cell lysis solution was added to lyse the cells, and the resulting cell lysate was transferred to a White Opaque 96-well Microplate (Perkin Elmer, Villebon-sur-Yvette, France) in preparation for the ATP assay. The intracellular ATP content was quantified using the ATP-lite assay kit (Perkin Elmer, Villebon-sur-Yvette, France). Luminescence intensity emanating from each well was measured using a FLUOstar Optima plate reader (BMG Labtech, Offenberg, Germany).

### 2.12 ROS measurement

Intracellular reactive oxygen species (ROS) levels were assessed using the mtSOX Deep Red (DOJINDO) method. Following the treatment of cells based on the experimental conditions, they were incubated with 10 μM mtSOX Deep Red for 30 min at 37 °C. Subsequently, the supernatant was discarded, and the cells were washed twice with HBSS and resuspended in HBSS buffer. Fluorescence signals were quantified using IN Cell Analyzer 2500HS (GE Healthcare).

### 2.13 Immunofluorescence staining

For fluorescence cytochemistry, cells were fixed in 4% paraformaldehyde for 15 min at room temperature on glass bottom dishes. Subsequently, the cells were rinsed three times with PBS for 5 min each and then incubated in a blocking buffer (0.3% Triton X-100 and 5% normal serum in PBS) for 60 min. Following the aspiration of the blocking solution, the samples were incubated overnight at 4 °C with the primary antibody diluted in antibody dilution buffer (0.3% Triton X-100 and 1% BSA in PBS). After rinsing three times with PBS, the samples were incubated in the dark at room temperature for 1 h with a fluorochrome-conjugated secondary antibody diluted in antibody dilution buffer. Nuclei were counterstained with Hoechst 33342 (1:500). Fluorescent images for all stained adipose tissue sections were captured with IN Cell Analyzer 2500HS (GE Healthcare). The antibodies used in this study are listed in Supplementary Table 2.

### 2.14 Histology

Adipose tissues obtained from male mice were fixed in 4% paraformaldehyde for 24 h and subsequently embedded in paraffin. To perform Hematoxylin and Eosin (H&E) or Sirius red staining, the paraffin blocks were sectioned into slides with a thickness of 3 μm, following standard protocols for H&E or Sirius red staining.

For immunohistochemistry, Anti-mCherry (1:200, Invitrogen, A32933) staining was conducted to detect tdTomato expression. Paraffin blocks were sectioned into slides with a thickness of 10 μm and underwent a series of steps, including deparaffinization in xylene and rehydration in 100%, 90%, and 70% alcohol. Antigen retrieval was performed by autoclaving at 120 °C for 20 min in citrate buffer (pH 6.0).

After pre-incubation with 10% goat serum albumin to block nonspecific sites at room temperature for 10 min, the sections were incubated overnight at 4 °C in a humidified chamber with the primary antibody against mCherry. Following washing, the sections were incubated with secondary antibodies and Hoechst and were then mounted with a fluorescence mounting medium. The fluorescent signals of the sections were captured using IN Cell Analyzer 2500HS (GE Healthcare). The antibodies used in this study are listed in Supplementary Table 2.

SA-b-gal staining was performed as previously described [35]. We utilized the BZ-X800 (Keyence) to capture images of stained cells post-staining. Fields of view were randomly selected, and the area of SA-β-gal staining was quantified in the images, and further normalized by cell area.

### 2.15 Seahorse respiration analysis

APCs were counted and seeded with 20,000 cells per well overnight in an Agilent Seahorse XFp cell culture microplate. The procedures used the In vitro Seahorse XF Cell Mito Stress Test Kit (Agilent) according to the manufacturer's instructions. The final concentrations of oligomycin, FCCP, rotenone, and antimycin-A were 15 μM, 20 μM, 5 μM, and 5 μM, respectively. The Agilent Seahorse XFp extracellular flux assay plate was inserted into XFp Extracellular Flux Analyzer (Seahorse Bioscience) to perform the analysis with standard protocol.

After the experiment, Hoechst dye was introduced into the wells for cell staining, and the number of cells per well was observed and quantified using a BZ-9000 analyzer (Keyence) and ImageJ software.

### 2.16 Statistics and reproducibility

For mouse experiments, all the male mice with the same genotype were randomly assigned to each group and independently followed the same age-dependent schedule in each experimental design. Sample sizes were not predetermined by pilot studies. The blinded designs were not performed in this study because of the automatic analyses obtained using the image analyzer with the same criteria.

GraphPad Prism was used for statistical analysis and graphs. Comparisons between the two groups were made by an unpaired two-tailed Student's t-test. Multiple comparisons of one-variable data were carried out by one-way analysis of variance (ANOVA) followed by the Tukey HSD test. p < 0.05 was considered to be statistically significant. The exact statistical parameters are shown in the figures. For all representative findings, triplicate or multiple independent experiments were performed, and similar results were obtained.

## 3 Results

### 3.1 p16^high^ cells localize predominantly to the stromal vascular fraction (SVF) in adipose tissue of aged mice

**Figure 1:**
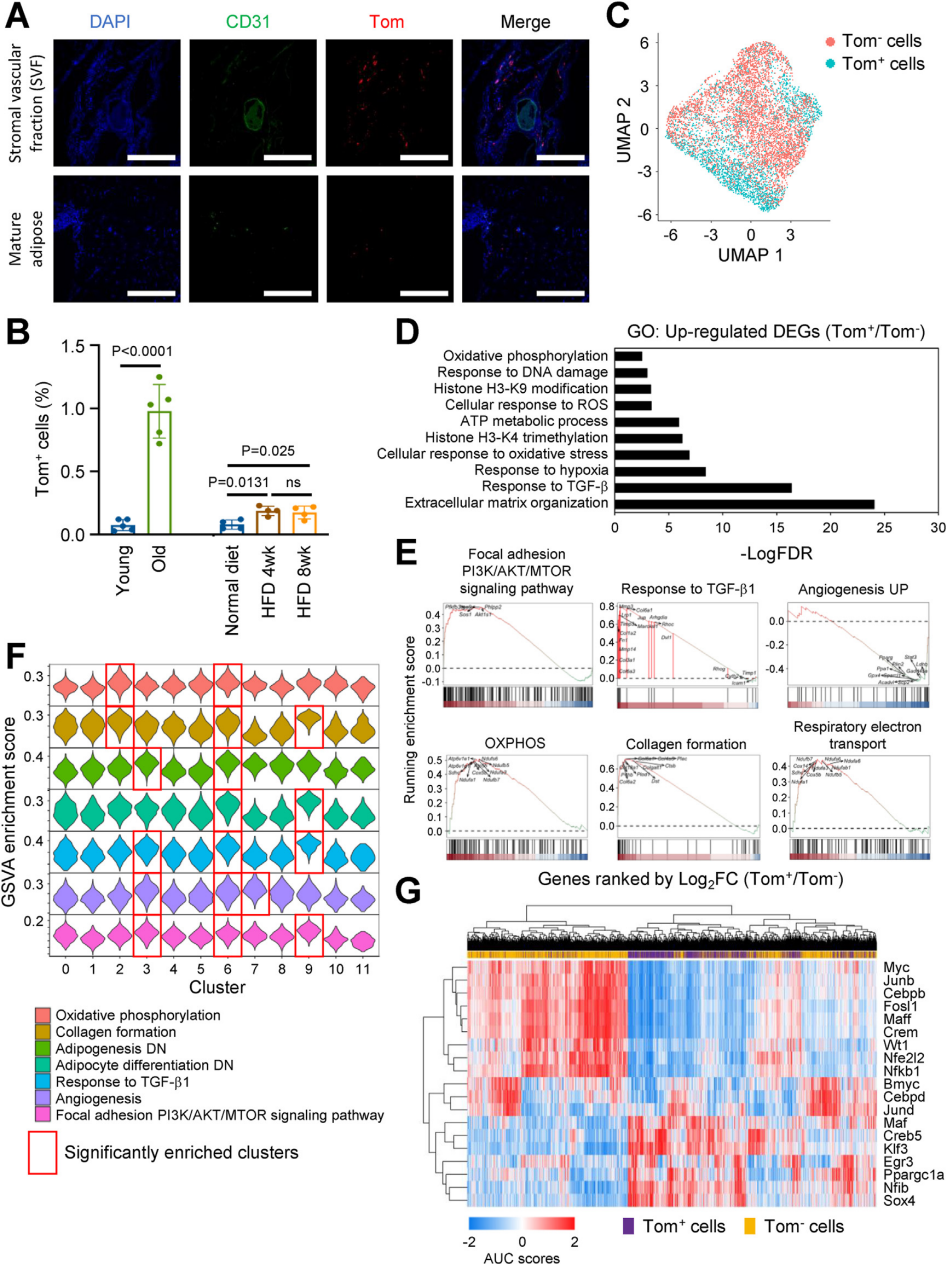
**scRNA-seq analysis reveals the loss of adipogenesis in p16^high^ APCs.A.** The immunofluorescence images of gWAT from 18-month-old p16-Tom mice using the indicated antibodies. Scale bar: 500 μm. **B.** The percentage of Tom^+^ APCs of gWAT quantified by FACS in young (3-month-old, n = 5), old (18-month-old, n = 5), normal diet (3-month-old, n = 4), 4 weeks HFD treated (3-month-old, n = 4), and 8 weeks HFD treated (3-month-old, n = 4) groups. All the mice were sacrificed 2 weeks after receiving 5 doses of TAM (80 mg/kgBW) daily via intraperitoneal injection. **C.** UMAP visualization of single-cell transcriptomes of Tom^+^ and Tom^−^ APCs isolated from old p16-Tom male mice (18-month-old). **D.** GO analysis results of up-regulated DEGs in Tom^+^ APCs compared to Tom^−^ APCs. All the GO terms were identified by FDR <0.05, and all the DEGs were qualified by Log_2_FC >0.35 and FDR <0.05. **E.** GSEA plots of indicated terms significantly enriched in the comparison between Tom^+^ APCs and Tom^−^ APCs. All the GSEA terms were determined by FDR <0.05. **F.** The violin plot showing the GSVA enrichment scores of indicated terms in each cluster. The red boxes indicate that the scores of this cluster were significantly higher than those of the other clusters. **G.** The heatmap showing the AUC scores of indicated transcription factors in each single cell transcriptome. Data are presented as means ± SEM of independent experiments **B**. Unpaired two-sided student's t-test (left panel of **B**) and one-way ANOVA with Tukey HSD test (right panel of **B** and **F**) were performed. FDR values were calculated by the B–H method.

Cellular senescence in adipose tissue is likely to cause multiple dysfunctions, including defective adipogenesis, inflammation, aberrant adipocytokine production, and insulin resistance. The reduced stemness and adipogenesis of aged APCs may also be a result of the accumulation of senescent cells. However, the molecular basis of senescence-induced adipose dysfunction remains largely unknown. To address this issue, we used mouse models in which p16^high^ cells can be visualized as tomato-positive cells, and isolated from different organs. We first investigated the *in vivo* dynamics of p16^high^ cells in gWAT during aging, as p16 is one of the most reliable senescent markers. In aged mice, we detected predominantly tomato-positive (Tom^+^) cells in the stromal vascular fraction (SVF), but not in the mature adipose region (Figure 1A). Cellular senescence of APCs and preadipocytes located in SVF [36] is considered one of the contributing factors to age-related dysfunction in adipose tissue [3]. In the section analysis, we found that Tom^+^ cells and PDGFRα^+^/Tom^+^ APCs [37] were significantly more abundant in the gWAT from old mice compared to young mice (Supplementary Fig. 1A and 1B). We then quantified the number of Tom^+^ cells by using FACS and found almost 20 times higher in aged SVF than that in young SVF (Figure 1B, Supplementary Fig. 1C). The up-regulation of p16 and other senescence markers such as p21, Glb1 [38], and one of the SASP factors, Igfbp5 [39], confirmed in Tom^+^ APCs (p16^high^ APCs) (Supplementary Fig. 1D). Strikingly, in obesity, the increase in the number of p16^high^ cells in the SVF was very marginal (Figure 1B). Single-cell RNA sequencing analysis (scRNA-seq) was performed to reveal the transcriptomic signatures of p16^high^ and p16^low^ adipose progenitor cells (APCs), excluding mature adipocytes (Figure 1C, Supplementary Fig. 2A).

APCs were classified into 12 clusters and p16^high^ cells were predominantly detected in clusters 2, 3, 6, and 9 (Supplementary Fig. 2B). The APC fraction also contains a preadipocyte population expressing Pparg (Supplementary Fig. 2C). The existence of Ly6C^+^ fibro-inflammatory progenitors (FIPs) [40], CD142^+^ adipose-regulatory cells (Aregs) [41], and Lgals3^+^ age-dependent regulatory cells (ARCs) [42] were identified by scRNA-seq techniques recently. In our scRNA-seq datasets, the expression levels of CD142 (shown as F3) and Lgals3 were similar between p16^high^ and p16^low^ APCs, while the expression of Ly6c1 was higher in p16^high^ APCs than in p16^low^ APCs (Supplementary Fig. 2D). Gene ontology (GO) analysis revealed that extracellular matrix organization, TGF-β signaling, and mitochondrial activation-related terms were enriched in upregulated differentially expressed genes (DEGs) in p16^high^ cells compared to that of p16^low^ cells (Figure 1D, Supplementary Fig. 2E). Gene set enrichment analysis (GSEA) also showed similar conclusions to the GO results (Figure 1E). Interestingly, adipogenesis-related genes were downregulated in p16^high^ APCs (Supplementary Fig. 2F). Consistent with this, gene set variation analysis (GSVA) revealed that the scores of extracellular matrix organization, TGF-β signaling, mitochondrial activation, and downregulation of adipogenesis-related terms were significantly high in the clusters 2, 3, 6, and 9, where p16^high^ cells were abundant (Figure 1F, Supplementary Fig. 2G). Gene regulatory network inference revealed that p16^high^ cells had low proliferative capacity (low Myc, Junb, and Fosl1) [43], and adipose differentiation potential (Cebpb and Sox4) [44,45] (Figure 1G).

### 3.2 M2 ATMs-derived TGF-β is involved in APC senescence *in vivo*

**Figure 2:**
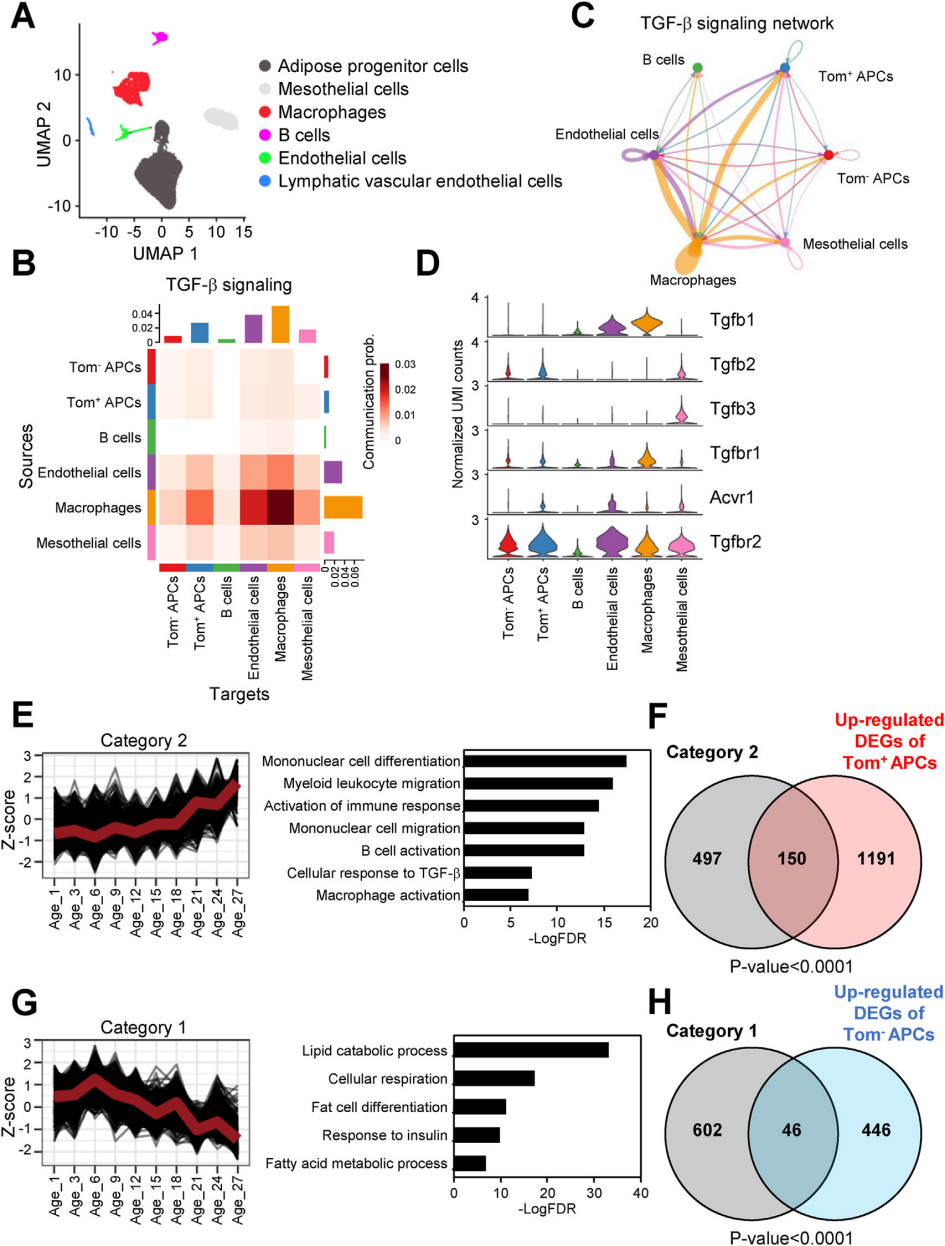
**scRNA-seq analysis implies the source of TGF-β signaling is ATM.A.** UMAP visualization of single-cell transcriptomes of gWAT cells isolated from old p16-Tom male mice (18-month-old). **B. and C.** The heatmap (**B**) and network plot (**C**) showing the ligand–receptor communication probabilities of TGF-β signaling between indicated cell types. **D.** Violin plots showing the expression levels of indicated cells in indicated cell types. **E.** The line graph depicting the average Z-score of genes in category 2 as they alter with the age of mice (left panel). The GO analysis results of genes in category 2 (right panel). **F.** The Venn diagram showing the intersection of genes in category 2 and up-regulated DEGs in Tom^+^ APC identified in scRNA-seq data. **G.** The line graph depicting the average Z-score of genes in category 1 as they alter with the age of mice (left panel). The GO analysis results of genes in category 1 (right panel). **H.** The Venn diagram showing the intersection of genes in category 1 and up-regulated DEGs in Tom^−^ APC identified in scRNA-seq data. All the GO terms were identified by FDR <0.05, and Fisher's exact test was performed to calculate the significance of the intersection (**F** and **H**). FDR values were calculated by the B–H method.

To investigate which signaling pathways induce senescence in APCs with age, we performed scRNA-seq analysis using sorted CD45^+^ cells and CD45^−^ cells from gWAT except for mature adipocytes (Figure 2A, Supplementary Fig. 3A). APCs were the most abundant cell types of Tom^+^ cells in CD45^−^ population (Supplementary Fig. 3B). The ligand–receptor interaction between each cell type revealed that downstream of TGF-β, PDGF, BMP, EGF, and IL6 signaling pathways were activated in p16^high^ APCs compared to p16^low^ cells (Supplementary Fig. 4A). Expression of ANGPTL, MK, MIF, PEIOSTIN, and non-canonical WNT signaling factors was upregulated in p16^high^ APCs compared to p16^low^ cells. Since p16^high^ APCs showed high expression of TGF-β-related genes (Figure 1D, E), we focused on the TGF-β signaling pathway. TGF-β was mainly derived from ATMs to p16^high^ APCs (Figure 2B, C). Consistently, the expression of Tgfbr2 was upregulated in the p16^high^ APCs (Figure 2D, Supplementary Fig. 4B). Extracellular matrix–receptor interaction revealed that collagen production and fibronectin were increased in p16^high^ APCs (Supplementary Fig. 4C). ATMs exhibit functional heterogeneity; M1 ATMs produce inflammatory cytokines and M2 have anti-inflammatory potential by secreting TGF-β. Indeed, scRNA-seq analysis revealed predominant expression of Arg1, a marker for M2, and marginal expression of Nos2, a marker for M1, suggesting that the majority of ATMs in aged mice are M2 (Supplementary Fig. 4D) [46]. The higher expression levels of Arg1 and Tgfb1 were confirmed in ATMs collected from the aged group compared to the young group (Supplementary Fig. 4E). On the other hand, Tnfa, pro-inflammatory cytokine and one of the M1 marker genes, were up-regulated in ATMs from obesity group. This result indicates that macrophages within aging adipose tissue exhibit different polarization states from obese adipose tissue.

To confirm that the transcriptional signature in p16^high^ APCs correlates with that of aging, we performed a time-series analysis of the published bulk RNA-seq dataset derived from WAT (Supplementary Fig. 5A) [30]. Highly variable genes were classified into 6 categories according to their time-dependent alteration patterns (Supplementary Fig. 5B). Among them, we noted that the average gene levels within category 2 showed a rise that was dependent on age. Genes related to TGF-β response and macrophage activation were significantly enriched in category 2 (Figure 2E). Genes found in category 2 displayed a significant overlap with upregulated DEGs in p16^high^ APCs (Figure 2F). Importantly, among the 26 genes related to the TGF-β response, 9 genes were shared by upregulated DEGs in p16^high^ APCs including Tgfbr2. Interestingly, we investigated 19 marker genes and found that determining M1 and M2 [47], and found 4 M2 marker genes such as CD115, CD206, CD163, and CD301, were enriched in the gene category 2. On the other hand, genes in category 1 showed an age-dependent decrease, and genes related to lipid catabolism and fat cell differentiation were enriched in this category (Figure 2G). Moreover, genes found in category 1 demonstrated an overlap with the downregulated DEGs in p16^high^ cells (Figure 2H).

Similar analyses were performed on human datasets from 20 to 79-year-old samples (Supplementary Fig. 6A) [33,34]. The highly variable genes were categorized into 6 categories, with category 4 displaying an age-dependent increase (Supplementary Fig. 6B–D). Notably, category 4 was enriched in response to TGF-β and genes related to macrophage activation exhibited a significant overlap with the upregulated DEGs in p16^high^ APCs (Supplementary Fig. 6E). In contrast, category 3 genes showed an age-dependent decrease and were enriched for genes related to protein folding and apoptosis (Supplementary Fig. 6F). These genes in category 3 overlapped with the downregulated DEGs in p16^high^ APCs (Supplementary Fig. 6G). The MHC-II antigen presentation-related terms were significantly enriched in genes belonging to another age-dependent decrease category 2, which suggested that the function of M1 macrophages likely declined with aging (Supplementary Fig. 6H).

### 3.3 TGF-β-induced senescence in APCs impairs adipogenesis

**Figure 3:**
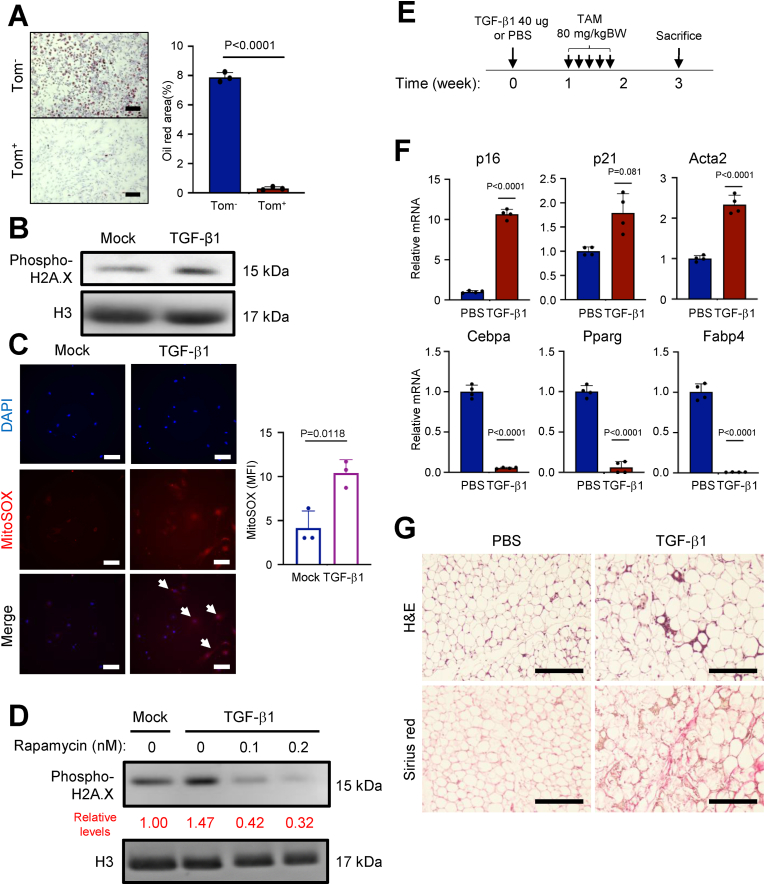
**TGF-β1 induces mitochondrial ROS and represses adipogenesis of APCs.A.** Quantitative results of Oil Red staining for Tom^+^ and Tom^−^ APCs isolated from 18-month-old p16-Tom male mice. The representative images were shown on the left panel. Scale bar: 100 μm. **B.** Immunoblotting of cell lysates with indicated antibodies. The APCs were treated by mock or 10 ng/ml TGF-β1 for 4 days. **C.** The MitoSOX staining of mitochondrial ROS for APCs with indicated treatments. The representative images were shown on the left panel. Scale bar: 100 μm. **D.** Immunoblotting of cell lysates with indicated antibodies. The APCs were treated by mock or 10 ng/ml TGF-β1 and rapamycin with indicated concentration for 4 days. The quantification of phospho-H2A.X was normalized against H3 and indicated in red text. **E.** The scheme of the TGF-β1 in situ injection experiment. All the male mice were 30-week-old and received one dose of PBS or 40 μg of TGF-β1 in gWAT. The administration of TAM was conducted daily for two weeks before the sacrifice. **F.** The relative mRNA levels of indicated genes for gWAT APCs sorted from mice treated as in **E**. **G.** The H&E (upper panel) and Sirius red (lower panel) staining images of gWAT from mice treated as in **E**. Scale bar: 200 μm. Data are presented as means ± SD of three or more independent experiments. An unpaired two-sided student's t-test (**A, C, and F**) was performed.

To investigate the effects of TGF-β on cellular senescence and adipogenesis in APCs, we isolated APCs from gWAT for subsequent *in vitro* experiments. APCs exhibit differentiation properties similar to mesenchymal stem cells (MSCs) [48], and TGF-β has been known to induce MSC differentiation into myofibroblasts [49]. To exclude the effects of myofibroblast differentiation from senescence, we divided the Sca-1^+^ APC population into DPP4^+^ and DPP4^−^ subsets followed by TGF-β1 treatment. We then found that the expression of myofibroblast marker, Acta2, was only up-regulated in DPP4^+^ cells but not in DPP4^−^ cells (Supplementary Fig. 7A). In our scRNA-seq data, Dpp4^+^ cells co-distributed with Ly6c1^+^ in the same population (Supplementary Fig. 2C), suggesting that DPP4^+^ cells may belong to the fibro-inflammatory progenitors (FIP) cell population [40,50]. Therefore, in our *in vitro* experiments, we focused on Sca-1^+^/DPP4^−^ APCs. To confirm the low adipogenic capacity of p16^high^ (Figure 1E), p16^low^ and p16^high^ APCs were isolated from gWAT of aged mice and differentiated into adipocytes *in vitro*. p16^high^ APCs showed lower adipocyte differentiation potential than p16^low^ APCs assessed by oil red staining (Figure 3A). We then investigated whether macrophage-derived TGF-β could induce senescent phenotypes in APCs (Figure 2B). TGF-β1 treated APCs from young and old mice showed upregulation of p16 and p21 and downregulation of adipogenesis-related genes (Supplementary Fig. 7B). Interestingly, old APCs were more sensitive to TGF-β1 for senescence induction, so APCs from aged mice were used thereafter. The SA-β-gal staining was conducted to confirm the senescent characteristics of TGF-β1 treated APCs (Supplementary Fig. 7C). A previous study suggested that a cell-autonomous effect of TGF-β on adipocytes regulates their metabolism [51]. We found that Tgfb1 and Tgfb2 showed positive feedback triggered by TGF-β1 stimulation in APCs (Supplementary Fig. 7D). TGF-β1 treatment reduced the adipogenic capacity of APCs in a dose-dependent manner (Supplementary Fig. 7E). To determine whether M2 macrophages could reduce adipogenic capacity and increase p16 expression of APCs, we co-cultured M2 bone marrow-derived macrophages (BMDMs) polarized by IL-4 treatment with APCs. We observed an increase in p16 expression and a reduction in adipogenesis-related genes 4 days after co-culture (Supplementary Fig. 7F).

TGF-β1 increased the amount of γH2AX and γH2AX-positive cells (Figure 3B, Supplementary Fig. 8A), suggesting that TGF-β1 may induce senescence of APCs due to DNA damage. Together with the observation that the response to ROS-related genes was upregulated in p16^high^ APCs (Figure 1D, Supplementary Fig. 8B), we examined the induction of ROS by TGF-β and found that TGF-β1 increased the level of mitochondrial ROS (Figure 3C), mitochondrial activity (Supplementary Fig. 8C), expression of mitochondrial genes (Supplementary Fig. 8D), and ATP production (Supplementary Fig. 8E). The mTOR inhibition by rapamycin could enhance mitophagy and rescue the DNA damage-induced senescence by TGF-β1 treatment (Figure 3D, Supplementary Fig. 8F).

Injection of TGF-β1 into gWAT of p16-tdTomato mice (Figure 3E) upregulated p16, p21, and Acta2 expression and downregulated adipogenesis-related genes in sorted APCs (Figure 3F). Consistent with this, the fibrotic area was increased in gWAT treated with TGF-β1 (Figure 3G). The body weight would not be affected by TGF-β1 injection (Supplementary Fig. 8G).

### 3.4 Cancer cachexia induces gWAT atrophy through APCs senescence

**Figure 4:**
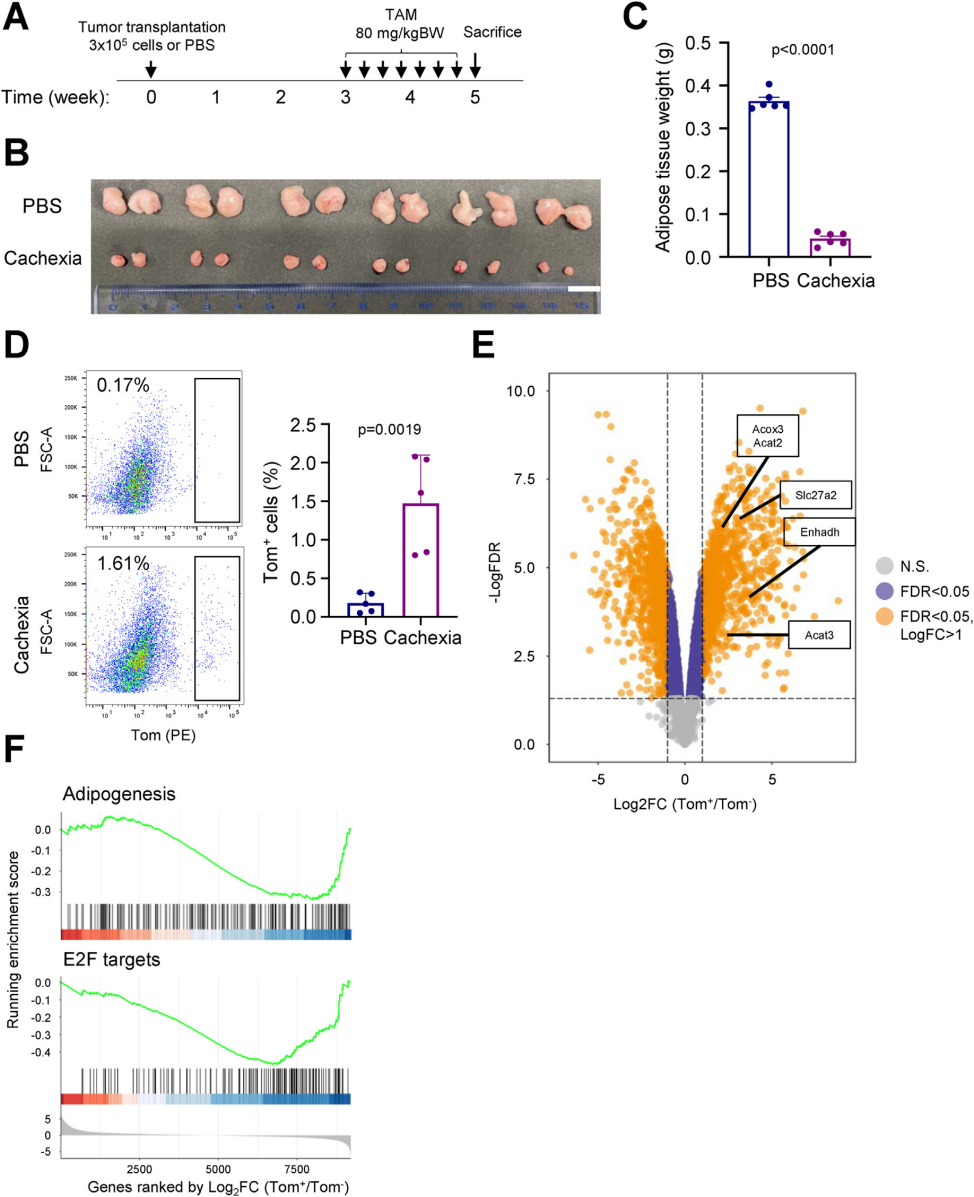
**The p16^high^ APCs correlate with lower adipogenesis ability in the cancer cachexia model.A.** The scheme of the allograft cancer cachexia model. All the p16-Tom male mice were 10-week-old and transplanted with PBS or 3 × 10^5^ PDAC cells subcutaneously. TAM injections were administered every two days, and a total of seven doses were given within the two weeks before the sacrifice. **B.** The pictures of gWAT collected from the mice treated as in **A**. Scale bar: 1 cm. **C.** The weight of the gWAT collected from the mice treated as in **A**. (n = 6 for each) **D.** The percentage of Tom^+^ APCs of gWAT quantified by FACS in PBS (n = 4), and cachexia (n = 5) groups. The representative scatter plots were shown in the left panel. **E.** The DEG analysis of bulk RNA-seq data between Tom^+^ (n = 3) and Tom^−^ (n = 3) APCs isolated from cachexia model of p16-Tom mice. All DEGs were determined by Log_2_FC >1 and FDR <0.05. Several genes related to lipid catabolism were labeled on the graph. **F.** The GSEA plots showing that indicated terms were significantly enriched in the Tom^−^ APCs compare to Tom^+^ APCs. Data are presented as means ± SD of three or more independent experiments. Unpaired two-sided student's t-test (**C** and **D**) was performed. FDR values were calculated by the B–H method.

WAT atrophy is one of the most typical phenotypes of cancer cachexia. Therefore, we asked whether APC senescence plays a role in this process. We subcutaneously transplanted mouse pancreatic ductal adenocarcinoma (PDAC) into p16-tdTomato mice (Figure 4A) and found that the size and weight of gWAT were dramatically reduced in cancer-bearing mice (Figure 4B, C), which were not caused by reduced food intake (Supplementary Fig. 9A). Importantly, we also found that the proportion of p16^high^ cells was increased in APCs (Figure 4D). Bulk RNA-seq analysis revealed that fatty acid consumption-related genes were upregulated in p16^high^ APCs compared to p16^low^ APCs (Figure 4E). GSEA showed that adipogenesis-related and E2F target genes were downregulated in p16^high^ APCs (Figure 4F).

However, we analyze the correlation of p16-dependent transcriptome alterations between aging scRNA-seq and cachexia bulk RNA-seq datasets. Although the adipogenesis-related genes were down-regulated in p16^high^ APCs from both datasets, the correlation was not significant (Supplementary Fig. 9B). We treated TGFBR inhibitor, SB431542 [[51], [52], [53]], in cachexia model and found that TGF-β blockade did not affect the population of p16^high^ APCs without the effects on food intake, adipose weight, and body weight (Supplementary Fig. 9C–9F). We confirmed that the ATMs collected from the cachexia model were M2 polarized as well (Arg1^+^). However, the dominant secreted cytokine of M2 ATMs derived from the cachexia model was Vegf but not Tgfb1 (Supplementary Fig. 9G), indicating that those belonged to the M2d subtype under the cancer cachexia [54]. This result suggests that p16^high^ APCs indeed have impaired adipogenic ability, but it might be caused by a different induction mechanism in the cachexia model from that in aging.

## 4 Discussion

Aging of adipose tissue alters several biological and physiological processes such as systemic glucose, lipid, and energy homeostasis. During aging, changes in the mass and redistribution of adipose tissue are common phenomena. Total fat mass increases in individuals as early as middle age and then decreases in old age. Adipose tissue is composed of not only adipocytes and APCs but also ATMs and vascular endothelial cells. Adipose tissue function and adipogenic capacity are regulated by their cross-talk but the precise mechanisms remain largely unknown. In addition, although obesity is considered to be a state of accelerated aging, its relationships concerning adipose function are also unknown.

Strikingly, in this study, we found that the population of senescent APCs was different between aging and obesity, showing a dramatic increase of the senescent APCs in aged but not obese gWAT. scRNA-seq analysis of aged gWAT revealed that macrophage-derived TGF-β induced senescence of APCs, which suppressed the adipogenic ability. Consistent with this, the majority of macrophages in aged gWAT polarized to the M2 state which was confirmed by upregulation of Arg1 and Tgfb1 in ATM. In this regard, previous studies showed that most macrophages in obese gWAT polarized to the M1 state [55,56]. M1 polarization was described to correlate with inflammation, and insulin resistance [57]. In contrast, M2 polarization was mentioned to be involved in lipid consumption and suppressing mitochondrial clearance [58], which was also observed in our setting. Therefore, the heterogeneity of ATMs in aged and obese gWAT likely determines the characteristics of APCs. A previous study showed that M1 macrophages are abundant in the aged WAT showing the high production of IL-6 [59]. The polarization of macrophages is highly heterogeneous and sensitive to the cytokine combination [60,61]. Rather than acute inflammation, it was reported that chronic inflammation positively correlated to the accumulation of M2 macrophages [62,63]. IL-6 is the typical marker of M1 macrophage. However, the pro-inflammatory cytokine, IL-6, has been described to enhance the sensitivity of macrophages to IL-4, which stimulates M2-polarization [64]. Indeed, M2-polarization and IL-10 expression were up-regulated in the lean-aged mice compared with middle-aged mice [65]. In this respect, it should be noted that aged animals used in the current study were mostly in the range of 30–35 g, which was considered as lean aged mice. Our results suggest that during the middle age, adipose expansion was accompanied by M1 polarization. In the lean-aged mice, M2 re-polarization caused by low-level chronic inflammation led to adipose atrophy through TGF-β1-dependent induction of APC senescence.

It has been recently reported that age-related accumulation of B cells [30] and their interaction with macrophage contributed to non-canonical lipolysis in adipose tissue [66]. In our scRNA-seq data, a part of B cells slightly expressed IL-10. The expanded B cell population with aging may be associated with M2 polarization. Although the effect of reprograming M2 macrophages in aged adipose tissue remains largely unknown, reprogramming tumor-associated M2 macrophages to anti-tumor M1 macrophages was expected to provide the therapeutic potential in the field of cancer study [67]. Targeting CSF1/CSF1R signaling might be a candidate to prevent M2 polarization, but there is no clear evidence demonstrating that CSF1 blockade treatment could redirect M2 to M0 or M1. Therefore, further research is needed to determine whether reprogramming M2 macrophages could improve age-related adipose atrophy and dysfunction.

TGF-β responses and macrophage activation are also common features of aging in both human and mouse WAT. Taken together, our results suggest that the polarization of M2 ATMs with age leads to a decline in adipogenic capacity through the induction of senescent APCs, resulting in the loss of fat mass in old age. Although senescent APCs have been reported to secrete activin A, which in turn suppresses the adipogenic potential of surrounding APCs, in our experimental condition, we did not detect an increase in the expression of activin A in senescent APCs, suggesting that the source of activin A was not senescent APCs.

The effect of TGF-β1 on the induction of APCs senescence and loss of adipogenic capacity was also confirmed *in vitro* [68]. Importantly, TGF-β1 treatment altered mitochondrial activity and oxidative stress through the induction of mitochondria-related genes. Dysregulation of mitochondrial activity has been proposed to induce senescence by producing high levels of ROS. Indeed, we observed high levels of mitochondrial ROS in TGF-β1 treated APCs. It has been reported that mTOR activity is required for mitochondrial biogenesis [69]. TGF-β signaling activates the mTOR pathway by activating the PI3K-AKT pathway [70]. As a positive feedback loop, mTOR activates SMAD2 and SMAD3, which further enhances TGF-β signaling [71].

Similar lipodystrophy is also a common feature of cancer-associated cachexia. A dramatic increase in the population of senescent APCs was also observed in tumor-bearing mice. Consistent with aged mice, senescent APCs showed a marked reduction in adipogenic capacity. However, bulk RNA-seq analysis did not show the activation of downstream TGF-β signaling in senescent APCs under cancer cachexia. Thus, other unidentified signaling pathways may be involved in the induction of senescent APCs in cachexic gWAT. Alternatively, systemic upregulation of TGF-β in the circulation may induce senescent APCs under cancer cachexia.

## CRediT authorship contribution statement

**Xinyi Zeng:** Writing – original draft, Methodology, Investigation, Formal analysis, Data curation. **Teh-Wei Wang:** Writing – original draft, Validation, Supervision, Methodology, Investigation, Formal analysis, Data curation, Conceptualization. **Kiyoshi Yamaguchi:** Investigation, Formal analysis, Data curation. **Seira Hatakeyama:** Investigation, Formal analysis. **Satoshi Yamazaki:** Resources. **Eigo Shimizu:** Formal analysis. **Seiya Imoto:** Formal analysis. **Yoichi Furukawa:** Formal analysis. **Yoshikazu Johmura:** Supervision, Funding acquisition. **Makoto Nakanishi:** Writing – review & editing, Supervision, Funding acquisition, Conceptualization.

## Declaration of competing interest

The authors declare the following financial interests/personal relationships which may be considered as potential competing interests: Makoto Nakanishi reports financial support was provided by 10.13039/501100001691Japan Society for the Promotion of Science. Makoto Nakanishi reports financial support was provided by 10.13039/100009619Japan Agency for Medical Research and Development. Yoshikazu Johmura reports financial support was provided by Japan Society for the Promotion of Science. Yoshikazu Johmura reports financial support was provided by Japan Agency for Medical Research and Development. Makoto Nakanishi reports a relationship with reverSASP Therapeutics that includes: consulting or advisory. Satoshi Yamazaki reports a relationship with Celaid Therapeutics that includes: consulting or advisory. If there are other authors, they declare that they have no known competing financial interests or personal relationships that could have appeared to influence the work reported in this paper.

## Data Availability

The scRNA-seq and bulk RNA-seq datasets described in this article are available in Gene Expression Omnibus (GEO) with accession number GSE264329, GSE263998, and GSE264000. The bulk RNA-seq analysis of public data were downloaded from GSE132040 and GTEx (v8) databases. All other data needed to evaluate the conclusions in the paper are presented in the paper and/or provided by corresponding authors.
