# Supplementary material for: M2 macrophage-derived TGF-β induces age-associated loss of adipogenesis through progenitor cell senescence
Source: Mol Metab. 2024 Apr 23;84:101943. doi: 10.1016/j.molmet.2024.101943 (PMC11079528; doi:10.1016/j.molmet.2024.101943)
Supplement: Multimedia component 1 [file mmc1.docx]

**Table S1 Primer lists**

| **Primers** | **Gene symbols** | **Sequences** |
| --- | --- | --- |
| qP-mmu-Fabp4-F | FABP4 | AAGGTGAAGAGCATCATAACCCT |
| qP-mmu-Fabp4-R | FABP4 | TCACGCCTTTCATAACACATTCC |
| qP-mmu-Pparg-F | PPARG | GCATGGTGCCTTCGCTGA |
| qP-mmu-Pparg-R | PPARG | TGGCATCTCTGTGTCAACCATG |
| qP-mmu-Cebpa-F | CEBPA | AAACAACGCAACGTGGAGA |
| qP-mmu-Cebpa-R | CEBPA | GCGGTCATTGTCACTGGTC |
| qP-mmu-Atp6-F | ATP6 | ACTTGCCCACTTCCTTCCACAA |
| qP-mmu-Atp6-R | ATP6 | TAAGCCGGACTGCTAATGCCA |
| qP-mmu-Cox3-F | COX3 | CTTCACCATCCTCCAAGCTTCA |
| qP-mmu-Cox3-R | COX3 | GTCCATGGAATCCAGTAGCCA |
| qP-mmu-Cox5b-F | COX5B | GCTGCATCTGTGAAGAGGACAAC |
| qP-mmu-Cox5b-R | COX5B | CAGCTTGTAATGGGTTCCACAGT |
| qP-mmu-Cycs-F | CYCS | CCAAATCTCCACGGTCTGTTC |
| qP-mmu-Cycs-R | CYCS | ATCAGGGTATCCTCTCCCCAG |
| qP-mmu-p16ink4a-F | p16ink4a | CCGCTGCAGACAGACTGG |
| qP-mmu-p16ink4a-R | p16ink4a | CCATCATCATCACCTGAATCG |
| qP-mmu-p21cip-F | p21cip | CCTGGTGATGTCCGAC |
| qP-mmu-p21cip-R | p21cip | CCATGAGCGCATCGCA |
| qP-mmu-Tfam-F | TFAM | TGGCTGAAGTTGGACGAAGT |
| qP-mmu-Tfam-R | TFAM | TGGGCCTAATCCCAATGACA |
| qP-mmu-Nrf1-F | NRF1 | ACCTTTGGAGAATGTGGTGCCA |
| qP-mmu-Nrf1-R | NRF1 | TGAATTAACCTCCTGTGGCGCA |
| qP-mmu-Acta2-F | ACTA2 | GTCCCAGACATCAGGGAGTAA |
| qP-mmu-Acta2-R | ACTA2 | TCGGATACTTCAGCGTCAGGA |
| qP-mmu-Arg1-F | ARG1 | CTCCAAGCCAAAGTCCTTAGAG |
| qP-mmu-Arg1-R | ARG1 | AGGAGCTGTCATTAGGGACATC |
| qP-mmu-Tnfa-F | TNFa | GGTGCCTATGTCTCAGCCTCTT |
| qP-mmu-Tnfa-R | TNFa | GCCATAGAACTGATGAGAGGGAG |
| qP-mmu-Vegf-F | VEGF | GCACATAGAGAGAATGAGCTTCC |
| qP-mmu-Vegf-R | VEGF | CTCCGCTCTGAACAAGGCT |
| qP-mmu-Igfbp5-F | IGFBP5 | AGACAGGAATCCGAACAAGGC |
| qP-mmu-Igfbp5-R | IGFBP5 | GTAGAATCCTTTGCGGTCACA |
| qP-mmu-Tgfbr1-F | TGFBR1 | TCTGCATTGCACTTATGCTGA |
| qP-mmu-Tgfbr1-R | TGFBR1 | AAAGGGCGATCTAGTGATGGA |
| qP-mmu-Tgfbr2-F | TGFBR2 | GACTGTCCACTTGCGACAAC |
| qP-mmu-Tgfbr2-R | TGFBR2 | GGCAAACCGTCTCCAGAGTAA |
| qP-mmu-Glb1-F | GLB1 | GCACGGCATCTATAATGTCACC |
| qP-mmu-Glb1-R | GLB1 | GTATCGGAATGGCTGTCCATC |
| qP-mmu-Tgfb1-F | TGFB1 | CTCCCGTGGCTTCTAGTGC |
| qP-mmu-Tgfb1-R | TGFB1 | GCCTTAGTTTGGACAGGATCTG |
| qP-mmu-Tgfb2-F | TGFB2 | TCGACATGGATCAGTTTATGCG |
| qP-mmu-Tgfb2-R | TGFB2 | CCCTGGTACTGTTGTAGATGGA |
| qP-mmu-bactin-F | ACTIN | GGCTGTATTCCCCTCCATCG |
| qP-mmu-bactin-R | ACTIN | CCAGTTGGTAACAATGCCATGT |

**Table S2 Antibodies**

| **For FACS** | | | |
| --- | --- | --- | --- |
| **Antibodies** | **Species** | **Catalog numbers** | **Suppliers** |
| APC Anti-mouse-CD31 | Rabbit | 102410 | BioLegend |
| APC Anti-mouse-CD45 | Rabbit | 103112 | BioLegend |
| BV605 Anti-mouse-Ly6A/E (Sca1) | Rabbit | 108134 | BioLegend |
| FITC Anti-mouse-CD26(DPP4) | Rabbit | 137805 | BioLegend |
| BV421 Anti-mouse-F4/80 | Rat | 123131 | BioLegend |
| PerCP/Cy5.5 Anti-mouse/human CD11b | Rat | 101228 | BioLegend |
| **For immunostaining** |  |  |  |
| **Antibodies** | **Species** | **Catalog numbers** | **Suppliers** |
| Anti-mouse-mCherry | Chicken | ab205402 | abcam |
| Anti-mouse-CD31 | Rabbit | ab222783 | abcam |
| Anti-mouse-Phospho-Histone H2A.X | Rabbit | 9718 | Cell Signaling Technology |
| Anti-mouse-CD140a | Rat | 14-1401-82 | Invitrogen |
| Anti-Chicken-IgG AF647 | Goat | A32933 | Invitrogen |
| Anti-Rabbit-IgG AF488 | Goat | ab150077 | abcam |
| Anti-Rabbit IgG AF647 | Donkey | ab150063 | abcam |

| **For immunoblotting** | | | |
| --- | --- | --- | --- |
| **Antibodies** | **Species** | **Catalog numbers** | **Suppliers** |
| Anti-mouse-Phospho-Histone H2A.X | Rabbit | 9718 | Cell Signaling Technology |
| Anti-mouse-Histone H3 | Mouse | 3638 | Cell Signaling Technology |
| Anti-mouse IgG HRP | Sheep | NA931 | Cyvita |
| Anti-rabbit IgG HRP | Donkey | NA934 | Cyvita |
