# Supplementary material for: M2 macrophage-derived TGF-β induces age-associated loss of adipogenesis through progenitor cell senescence
Source: Mol Metab. 2024 Apr 23;84:101943. doi: 10.1016/j.molmet.2024.101943 (PMC11079528; doi:10.1016/j.molmet.2024.101943)
Supplement: Multimedia component 2 [file mmc2.pptx]

## Slide 1
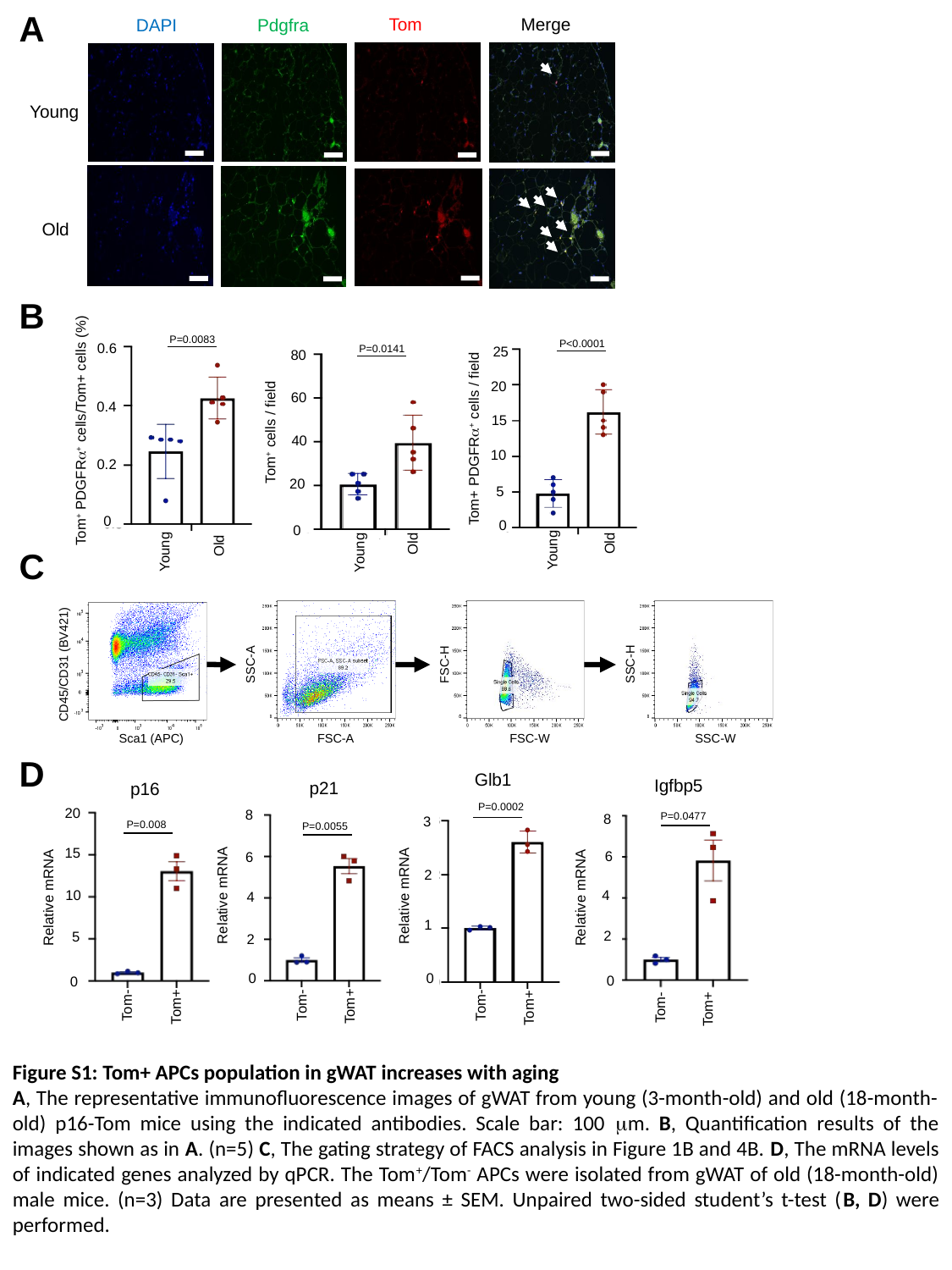

A
Tom
Merge
Pdgfra
DAPI
Young
Old
B
P=0.0083
0.6
0.4
Tom+ PDGFRa+ cells/Tom+ cells (%)
0.2
0
Old
Young
P=0.0141
60
Tom+ cells / field
40
20
0
Old
Young
P<0.0001
15
Tom+ PDGFRa+ cells / field
10
5
0
Old
Young
25
20
80
C
CD45/CD31 (BV421)
SSC-A
FSC-H
SSC-H
Sca1 (APC)
FSC-A
FSC-W
SSC-W
D
Glb1
P=0.0002
3
2
Relative mRNA
1
0
Tom+
Tom-
P=0.0055
6
Relative mRNA
4
2
0
Tom+
Tom-
p21
8
Igfbp5
P=0.0477
6
4
Relative mRNA
2
0
Tom+
Tom-
8
P=0.008
15
10
Relative mRNA
5
0
Tom+
Tom-
p16
20
Figure S1: Tom+ APCs population in gWAT increases with aging
A, The representative immunofluorescence images of gWAT from young (3-month-old) and old (18-month-old) p16-Tom mice using the indicated antibodies. Scale bar: 100 mm. B, Quantification results of the images shown as in A. (n=5) C, The gating strategy of FACS analysis in Figure 1B and 4B. D, The mRNA levels of indicated genes analyzed by qPCR. The Tom+/Tom- APCs were isolated from gWAT of old (18-month-old) male mice. (n=3) Data are presented as means ± SEM. Unpaired two-sided student’s t-test (B, D) were performed.

## Slide 2
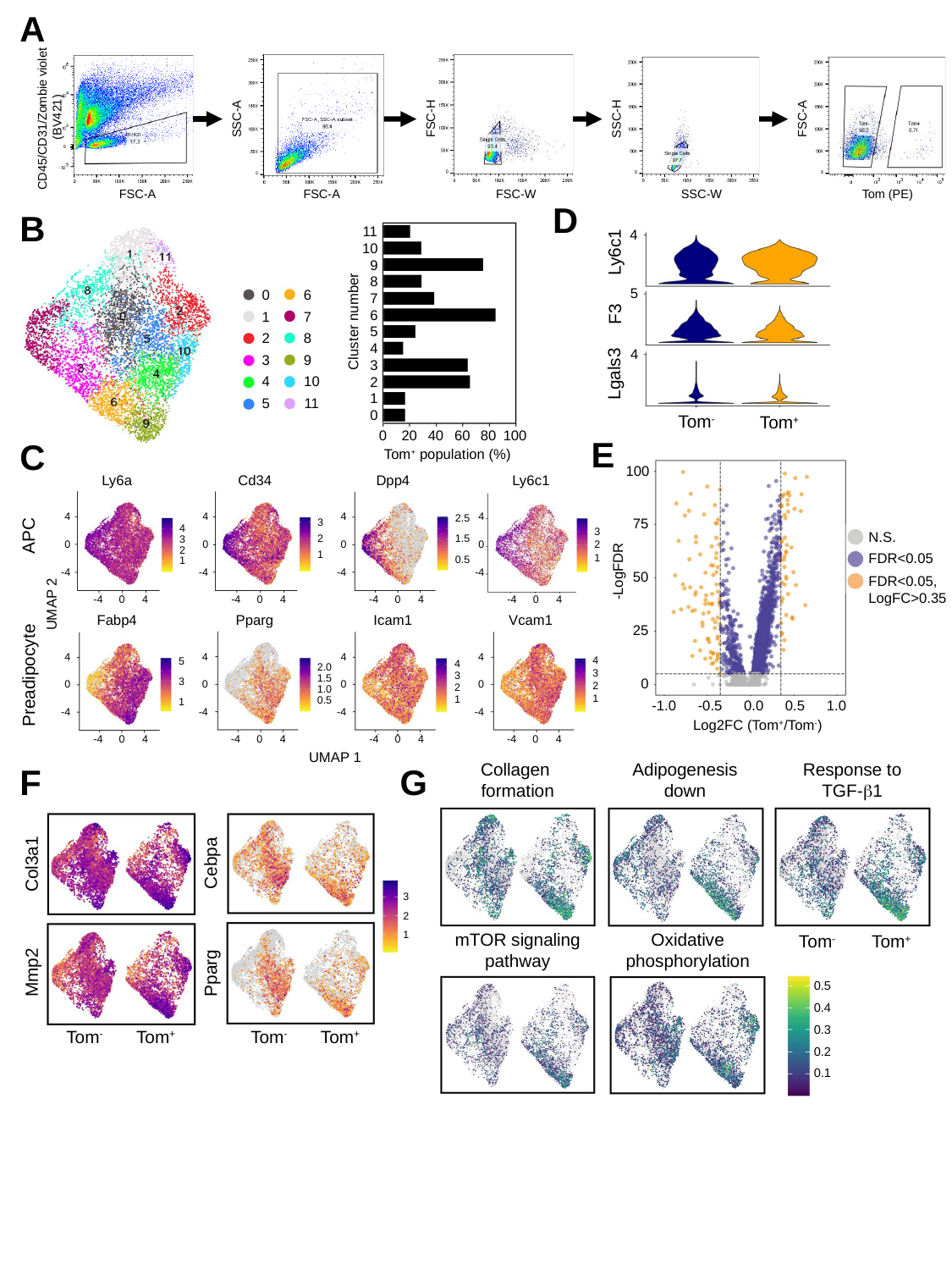

A
CD45/CD31/Zombie violet
 (BV421)
SSC-A
FSC-H
SSC-H
FSC-A
FSC-A
FSC-A
FSC-W
SSC-W
Tom (PE)
D
B
4
Ly6c1
5
F3
4
Lgals3
Tom-
Tom+
11
10
9
8
7
6
Cluster number
5
4
3
2
1
0
0
20
40
60
80
100
Tom+ population (%)
0
6
1
7
2
8
3
9
4
10
5
11
E
C
100
Ly6a
4
0
-4
-4
0
4
Cd34
4
0
-4
-4
0
4
Dpp4
4
0
-4
-4
0
4
Ly6c1
4
0
-4
-4
0
4
2.5
1.5
0.5
3
2
1
4
3
2
1
3
2
1
APC
UMAP 2
Fabp4
4
0
-4
-4
0
4
Pparg
4
0
-4
-4
0
4
Icam1
4
0
-4
-4
0
4
Vcam1
4
0
-4
-4
0
4
4
3
2
1
5
3
1
4
3
2
1
2.0
1.5
1.0
0.5
Preadipocyte
UMAP 1
75
N.S.
FDR<0.05
-LogFDR
50
FDR<0.05,
LogFC>0.35
25
0
-1.0
-0.5
0.0
0.5
1.0
Log2FC (Tom+/Tom-)
Collagen
formation
Adipogenesis
down
Response to TGF-b1
mTOR signaling pathway
Oxidative phosphorylation
Tom-
Tom+
0.5
0.4
0.3
0.2
0.1
F
G
Cebpa
Col3a1
3
2
1
Mmp2
Pparg
Tom-
Tom+
Tom-
Tom+

## Slide 3
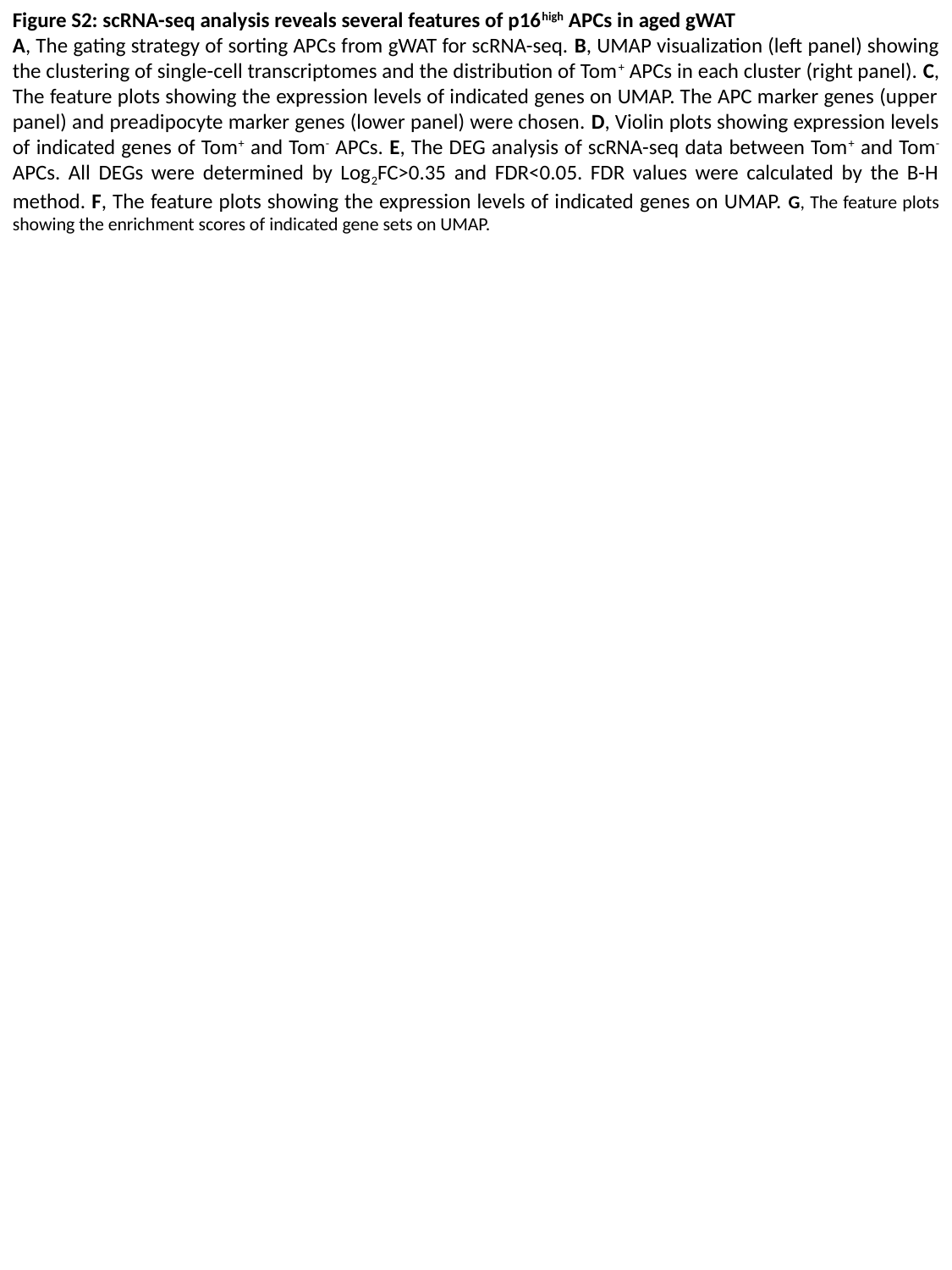

Figure S2: scRNA-seq analysis reveals several features of p16high APCs in aged gWAT
A, The gating strategy of sorting APCs from gWAT for scRNA-seq. B, UMAP visualization (left panel) showing the clustering of single-cell transcriptomes and the distribution of Tom+ APCs in each cluster (right panel). C, The feature plots showing the expression levels of indicated genes on UMAP. The APC marker genes (upper panel) and preadipocyte marker genes (lower panel) were chosen. D, Violin plots showing expression levels of indicated genes of Tom+ and Tom- APCs. E, The DEG analysis of scRNA-seq data between Tom+ and Tom- APCs. All DEGs were determined by Log2FC>0.35 and FDR<0.05. FDR values were calculated by the B-H method. F, The feature plots showing the expression levels of indicated genes on UMAP. G, The feature plots showing the enrichment scores of indicated gene sets on UMAP.

## Slide 4
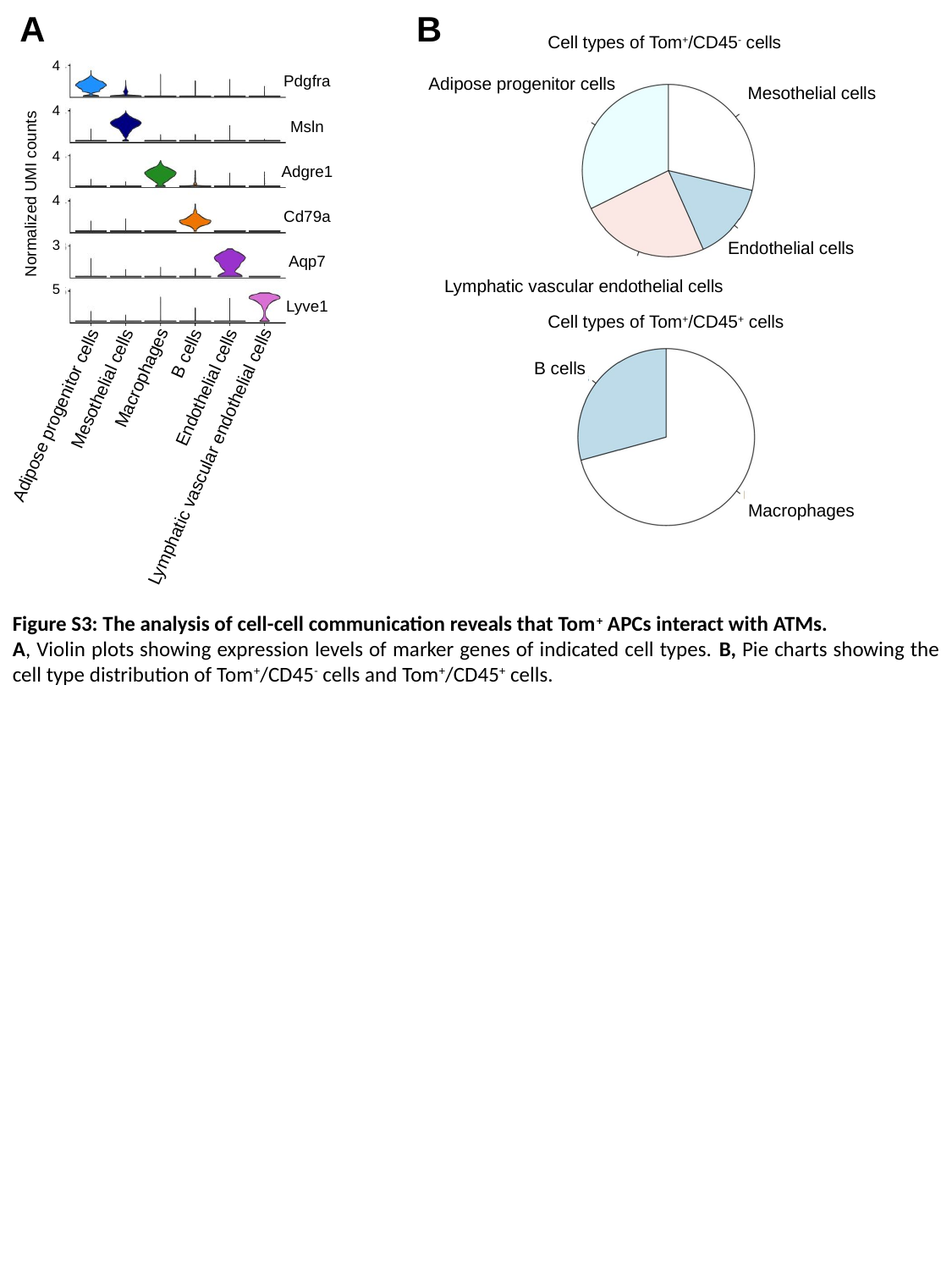

A
B
Cell types of Tom+/CD45- cells
4
Pdgfra
4
Msln
4
Adgre1
Normalized UMI counts
4
Cd79a
3
Aqp7
5
Lyve1
B cells
Macrophages
Endothelial cells
Mesothelial cells
Adipose progenitor cells
Lymphatic vascular endothelial cells
Adipose progenitor cells
Mesothelial cells
Endothelial cells
Lymphatic vascular endothelial cells
Cell types of Tom+/CD45+ cells
B cells
Macrophages
Figure S3: The analysis of cell-cell communication reveals that Tom+ APCs interact with ATMs.
A, Violin plots showing expression levels of marker genes of indicated cell types. B, Pie charts showing the cell type distribution of Tom+/CD45- cells and Tom+/CD45+ cells.

## Slide 5
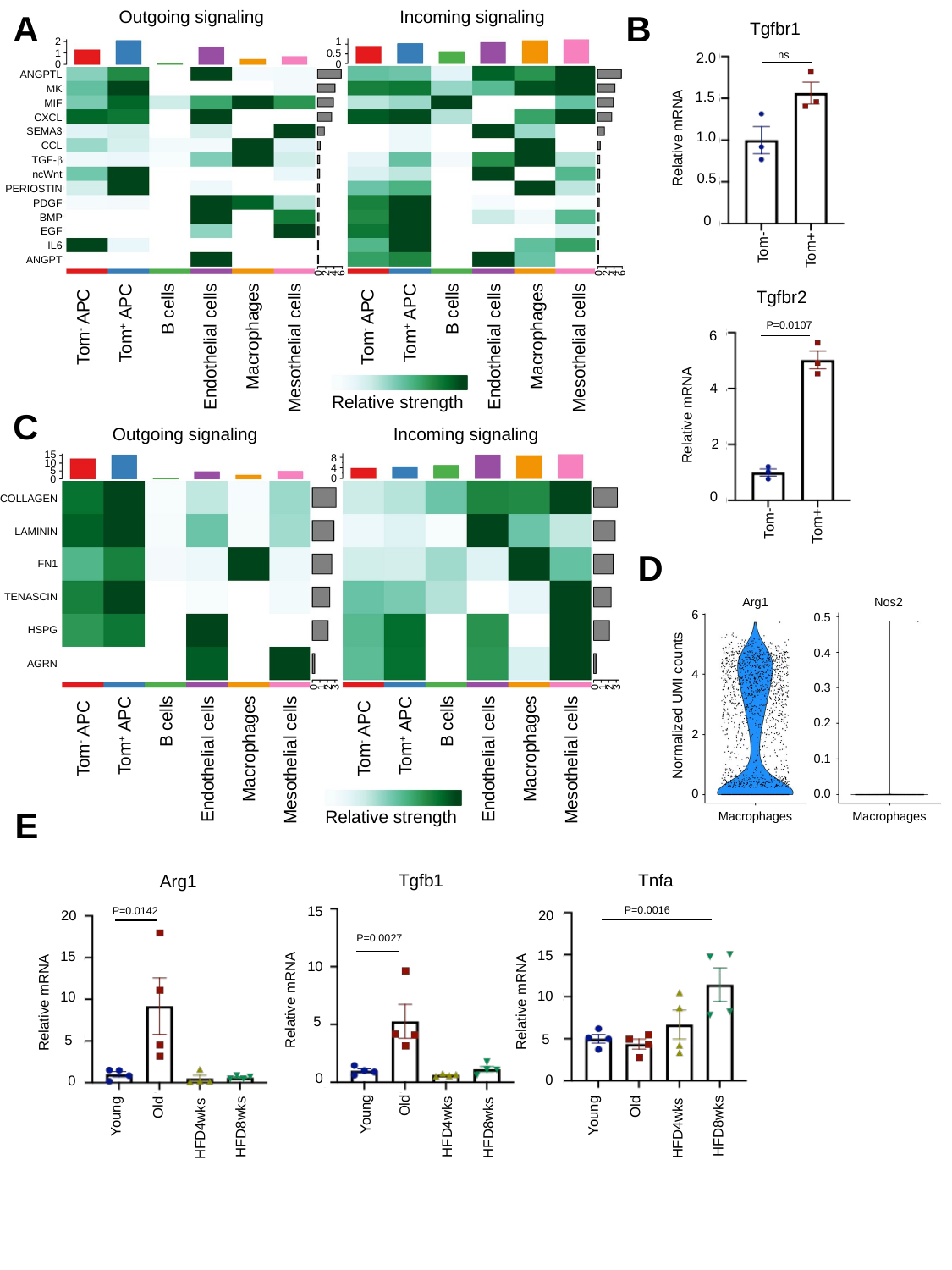

A
B
Outgoing signaling
Incoming signaling
2
1
1
0.5
0
0
ANGPTL
MK
MIF
CXCL
SEMA3
CCL
TGF-b
ncWnt
PERIOSTIN
PDGF
BMP
EGF
IL6
ANGPT
0
2
4
6
0
2
4
6
B cells
B cells
Tom+ APC
Tom+ APC
Tom- APC
Tom- APC
Macrophages
Macrophages
Endothelial cells
Endothelial cells
Mesothelial cells
Mesothelial cells
Relative strength
ns
1.5
1.0
Relative mRNA
0.5
0
Tom+
Tom-
Tgfbr1
2.0
Tgfbr2
P=0.0107
6
4
Relative mRNA
2
0
Tom+
Tom-
C
Outgoing signaling
Incoming signaling
15
8
10
4
5
0
0
COLLAGEN
LAMININ
FN1
TENASCIN
HSPG
AGRN
0
1
2
3
0
1
2
3
B cells
B cells
Tom+ APC
Tom+ APC
Tom- APC
Tom- APC
Macrophages
Macrophages
Endothelial cells
Endothelial cells
Mesothelial cells
Mesothelial cells
Relative strength
D
Arg1
6
4
Normalized UMI counts
2
0
Macrophages
Nos2
0.5
0.4
0.3
0.2
0.1
0.0
Macrophages
E
Tgfb1
15
P=0.0027
10
Relative mRNA
5
0
Old
Young
HFD4wks
HFD8wks
Tnfa
P=0.0016
20
15
10
Relative mRNA
5
0
Old
HFD8wks
Young
HFD4wks
Arg1
P=0.0142
20
15
10
Relative mRNA
5
0
Old
HFD8wks
Young
HFD4wks

## Slide 6
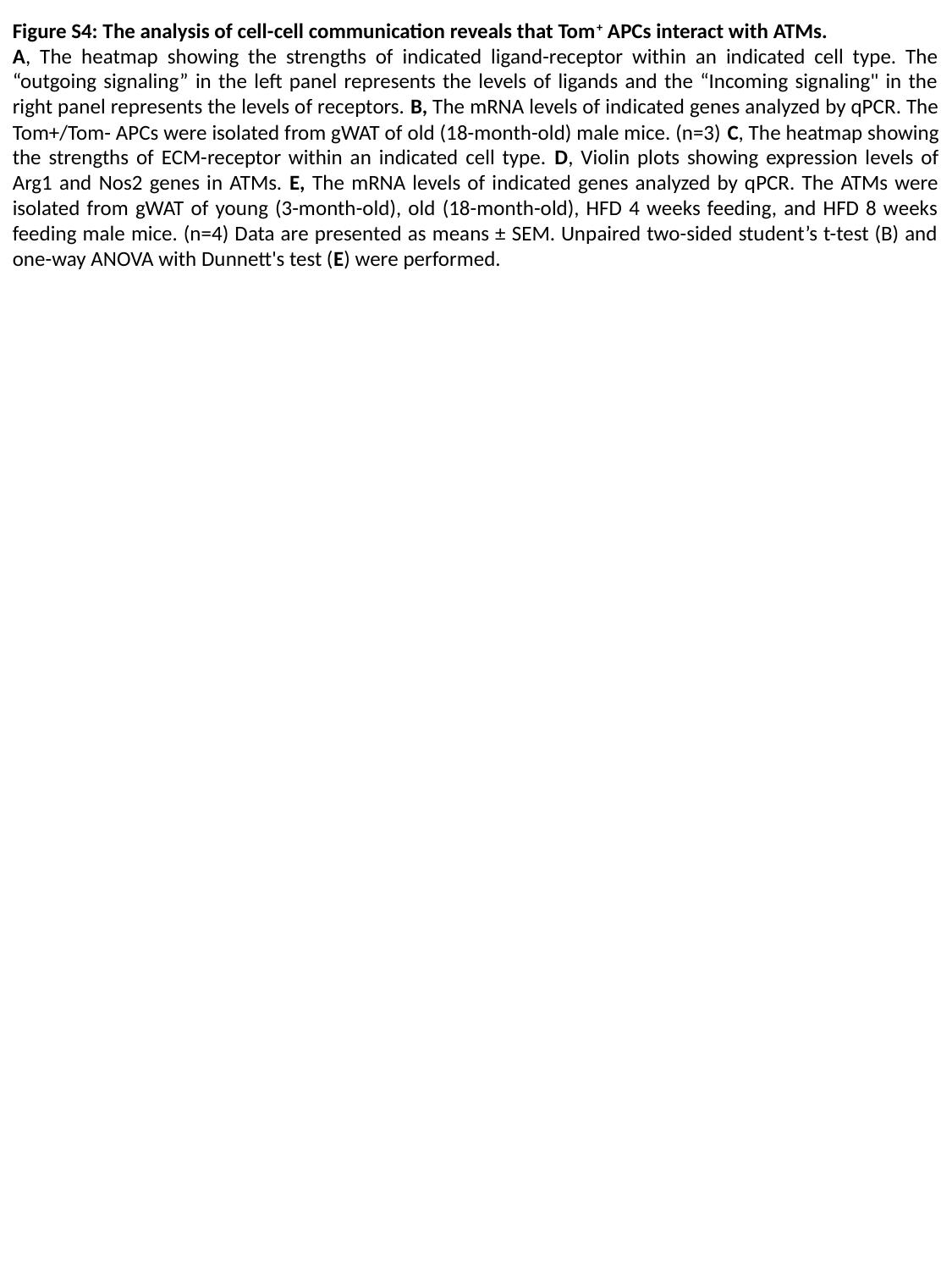

Figure S4: The analysis of cell-cell communication reveals that Tom+ APCs interact with ATMs.
A, The heatmap showing the strengths of indicated ligand-receptor within an indicated cell type. The “outgoing signaling” in the left panel represents the levels of ligands and the “Incoming signaling" in the right panel represents the levels of receptors. B, The mRNA levels of indicated genes analyzed by qPCR. The Tom+/Tom- APCs were isolated from gWAT of old (18-month-old) male mice. (n=3) C, The heatmap showing the strengths of ECM-receptor within an indicated cell type. D, Violin plots showing expression levels of Arg1 and Nos2 genes in ATMs. E, The mRNA levels of indicated genes analyzed by qPCR. The ATMs were isolated from gWAT of young (3-month-old), old (18-month-old), HFD 4 weeks feeding, and HFD 8 weeks feeding male mice. (n=4) Data are presented as means ± SEM. Unpaired two-sided student’s t-test (B) and one-way ANOVA with Dunnett's test (E) were performed.

## Slide 7
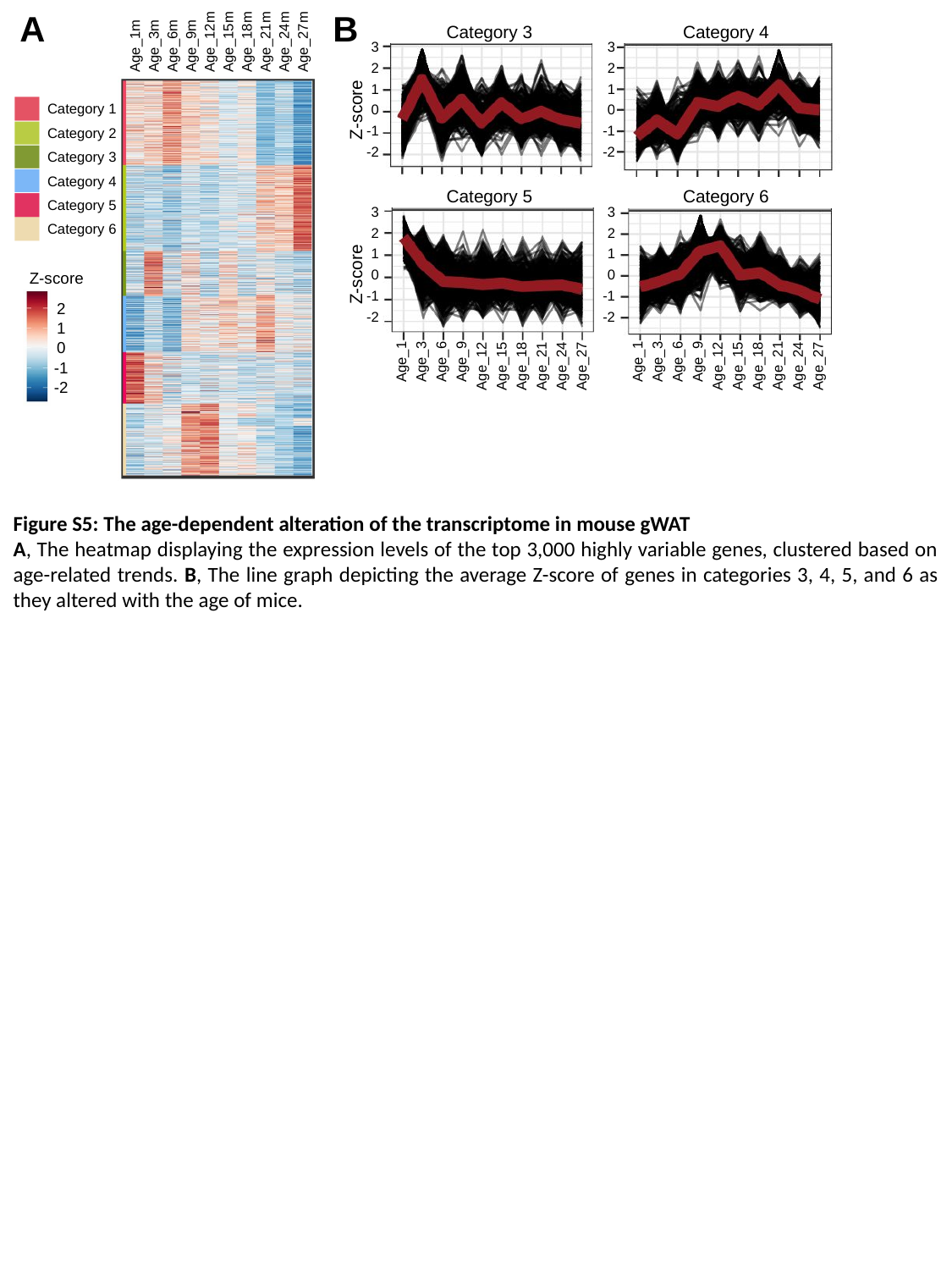

A
B
Category 3
Category 4
3
3
2
2
1
1
Z-score
0
0
-1
-1
-2
-2
Category 5
Category 6
3
3
2
2
1
1
Z-score
0
0
-1
-1
-2
-2
Age_1
Age_3
Age_6
Age_9
Age_12
Age_15
Age_18
Age_21
Age_24
Age_27
Age_1
Age_3
Age_6
Age_9
Age_12
Age_15
Age_18
Age_21
Age_24
Age_27
Age_12m
Age_15m
Age_18m
Age_21m
Age_24m
Age_27m
Age_1m
Age_3m
Age_6m
Age_9m
Category 1
Category 2
Category 3
Category 4
Category 5
Category 6
Z-score
2
1
0
-1
-2
Figure S5: The age-dependent alteration of the transcriptome in mouse gWAT
A, The heatmap displaying the expression levels of the top 3,000 highly variable genes, clustered based on age-related trends. B, The line graph depicting the average Z-score of genes in categories 3, 4, 5, and 6 as they altered with the age of mice.

## Slide 8
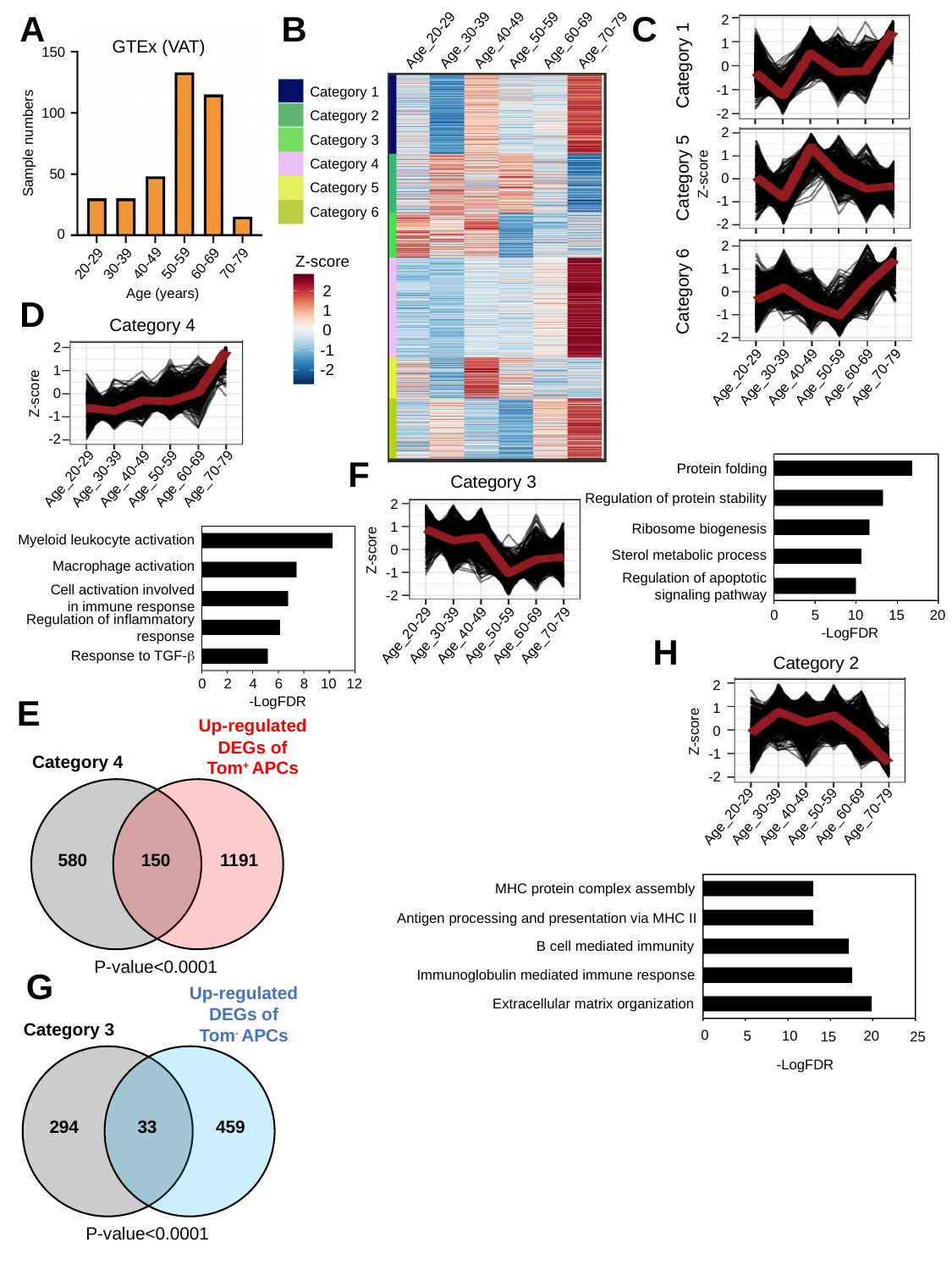

A
B
C
Age_20-29
Age_30-39
Age_40-49
Age_50-59
Age_60-69
Age_70-79
Category 1
Category 2
Category 3
Category 4
Category 5
Category 6
Z-score
2
1
0
-1
-2
2
GTEx (VAT)
150
100
Sample numbers
50
0
20-29
30-39
40-49
50-59
60-69
70-79
Age (years)
1
Category 1
0
-1
-2
2
1
Z-score
Category 5
0
-1
-2
2
1
Category 6
0
D
-1
Category 4
2
1
0
Z-score
-1
-2
Age_20-29
Age_30-39
Age_40-49
Age_50-59
Age_60-69
Age_70-79
-2
Age_20-29
Age_30-39
Age_40-49
Age_50-59
Age_60-69
Age_70-79
F
Protein folding
Regulation of protein stability
Ribosome biogenesis
Sterol metabolic process
Regulation of apoptotic signaling pathway
0
5
10
15
20
-LogFDR
Category 3
2
1
0
Z-score
-1
-2
Age_20-29
Age_30-39
Age_40-49
Age_50-59
Age_60-69
Age_70-79
Myeloid leukocyte activation
Macrophage activation
Cell activation involved
 in immune response
Regulation of inflammatory
 response
Response to TGF-b
0
2
4
6
8
10
12
-LogFDR
H
Category 2
2
1
0
Z-score
-1
-2
Age_20-29
Age_30-39
Age_40-49
Age_50-59
Age_60-69
Age_70-79
E
Up-regulated DEGs of Tom+ APCs
Category 4
580
150
1191
P-value<0.0001
MHC protein complex assembly
Antigen processing and presentation via MHC II
B cell mediated immunity
Immunoglobulin mediated immune response
Extracellular matrix organization
0
5
10
20
15
25
-LogFDR
G
Up-regulated DEGs of Tom- APCs
Category 3
294
33
459
P-value<0.0001

## Slide 9
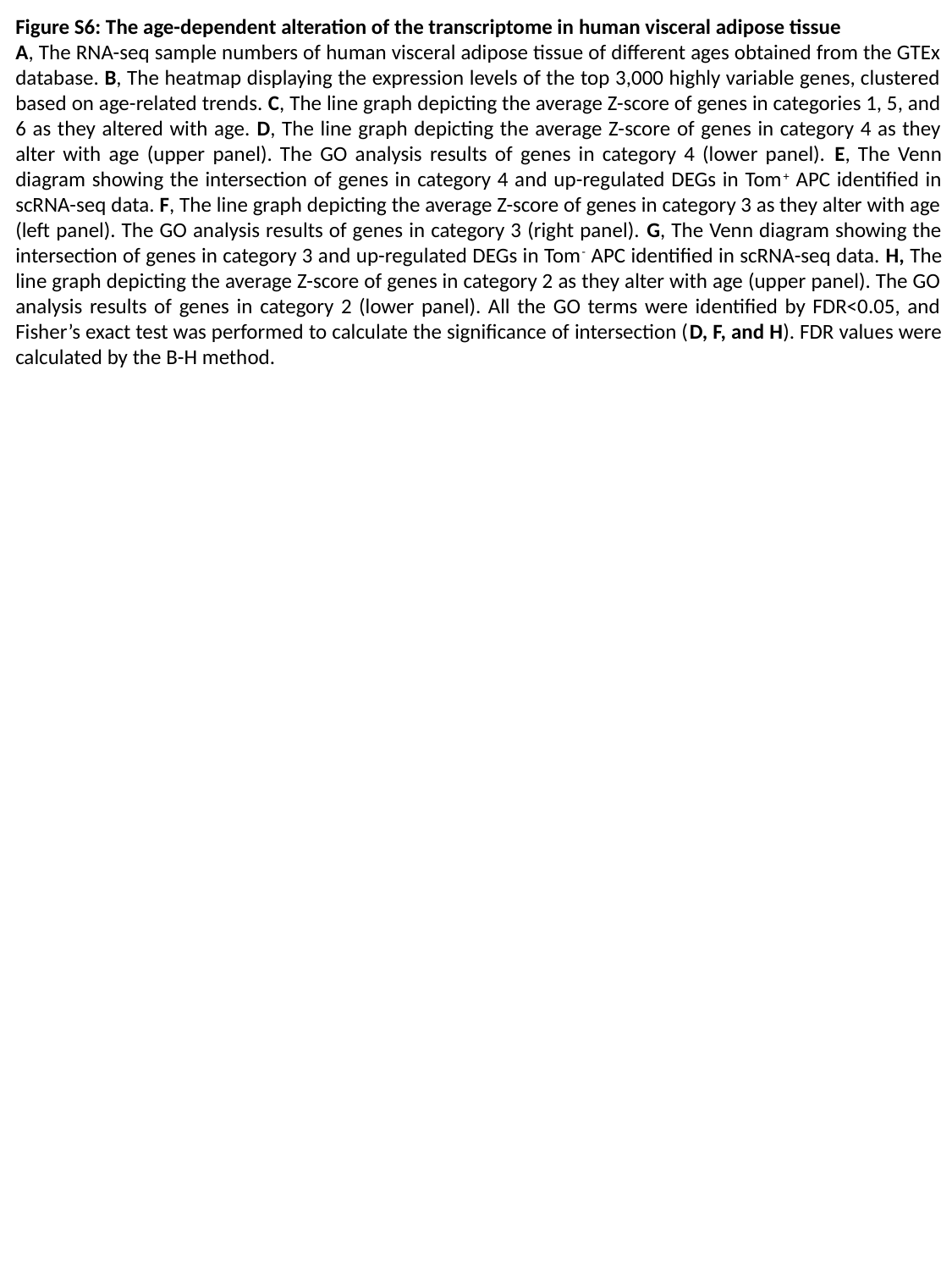

Figure S6: The age-dependent alteration of the transcriptome in human visceral adipose tissue
A, The RNA-seq sample numbers of human visceral adipose tissue of different ages obtained from the GTEx database. B, The heatmap displaying the expression levels of the top 3,000 highly variable genes, clustered based on age-related trends. C, The line graph depicting the average Z-score of genes in categories 1, 5, and 6 as they altered with age. D, The line graph depicting the average Z-score of genes in category 4 as they alter with age (upper panel). The GO analysis results of genes in category 4 (lower panel). E, The Venn diagram showing the intersection of genes in category 4 and up-regulated DEGs in Tom+ APC identified in scRNA-seq data. F, The line graph depicting the average Z-score of genes in category 3 as they alter with age (left panel). The GO analysis results of genes in category 3 (right panel). G, The Venn diagram showing the intersection of genes in category 3 and up-regulated DEGs in Tom- APC identified in scRNA-seq data. H, The line graph depicting the average Z-score of genes in category 2 as they alter with age (upper panel). The GO analysis results of genes in category 2 (lower panel). All the GO terms were identified by FDR<0.05, and Fisher’s exact test was performed to calculate the significance of intersection (D, F, and H). FDR values were calculated by the B-H method.

## Slide 10
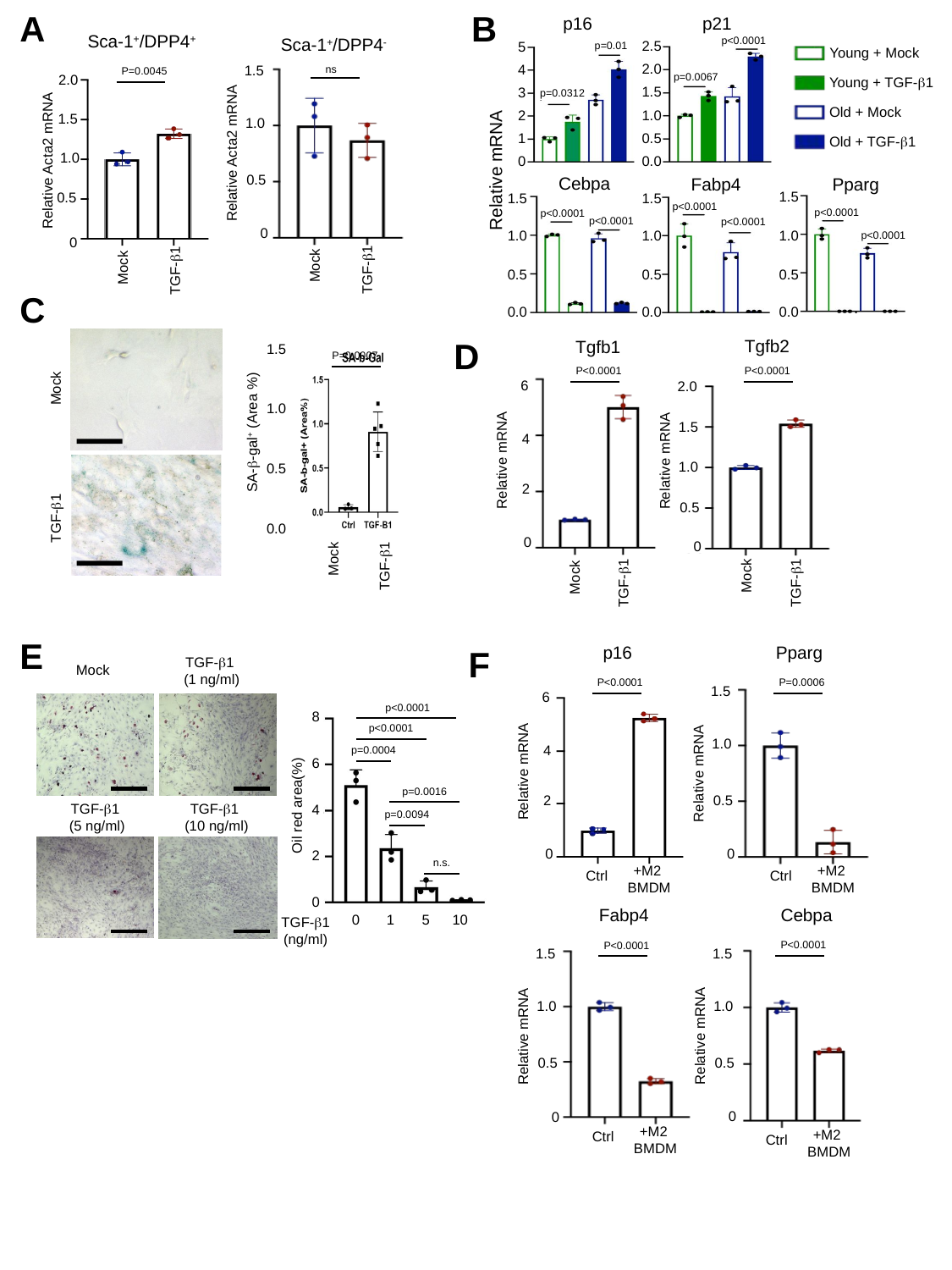

A
B
p16
p21
p<0.0001
2.5
5
p=0.01
2.0
4
p=0.0067
1.5
3
p=0.0312
1.0
2
0.5
1
0
0.0
Relative mRNA
Cebpa
Fabp4
Pparg
1.5
1.5
1.5
p<0.0001
p<0.0001
p<0.0001
p<0.0001
p<0.0001
1.0
1.0
1.0
p<0.0001
0.5
0.5
0.5
0.0
0.0
0.0
Young + Mock
Young + TGF-b1
Old + Mock
Old + TGF-b1
Sca-1+/DPP4+
P=0.0045
1.5
1.0
Relative Acta2 mRNA
0.5
0
Mock
TGF-b1
2.0
1.5
ns
1.0
Relative Acta2 mRNA
0.5
0
Mock
TGF-b1
Sca-1+/DPP4-
C
D
Tgfb2
P<0.0001
2.0
1.5
Relative mRNA
1.0
0.5
0
TGF-b1
Mock
Tgfb1
P<0.0001
6
4
Relative mRNA
2
0
TGF-b1
Mock
1.5
P=0.0007
1.0
SA-b-gal+ (Area %)
0.5
0.0
TGF-b1
Mock
Mock
TGF-b1
E
p16
P<0.0001
6
4
Relative mRNA
2
0
+M2
BMDM
Ctrl
Pparg
P=0.0006
1.5
1.0
Relative mRNA
0.5
0
+M2
BMDM
Ctrl
Cebpa
P<0.0001
1.5
1.0
Relative mRNA
0.5
0
Fabp4
P<0.0001
1.5
1.0
Relative mRNA
0.5
0
+M2
BMDM
+M2
BMDM
Ctrl
Ctrl
F
TGF-b1
(1 ng/ml)
Mock
TGF-b1
(5 ng/ml)
TGF-b1
(10 ng/ml)
p<0.0001
8
p<0.0001
p=0.0004
6
p=0.0016
Oil red area(%)
4
p=0.0094
2
n.s.
0
0
1
5
10
TGF-b1
(ng/ml)

## Slide 11
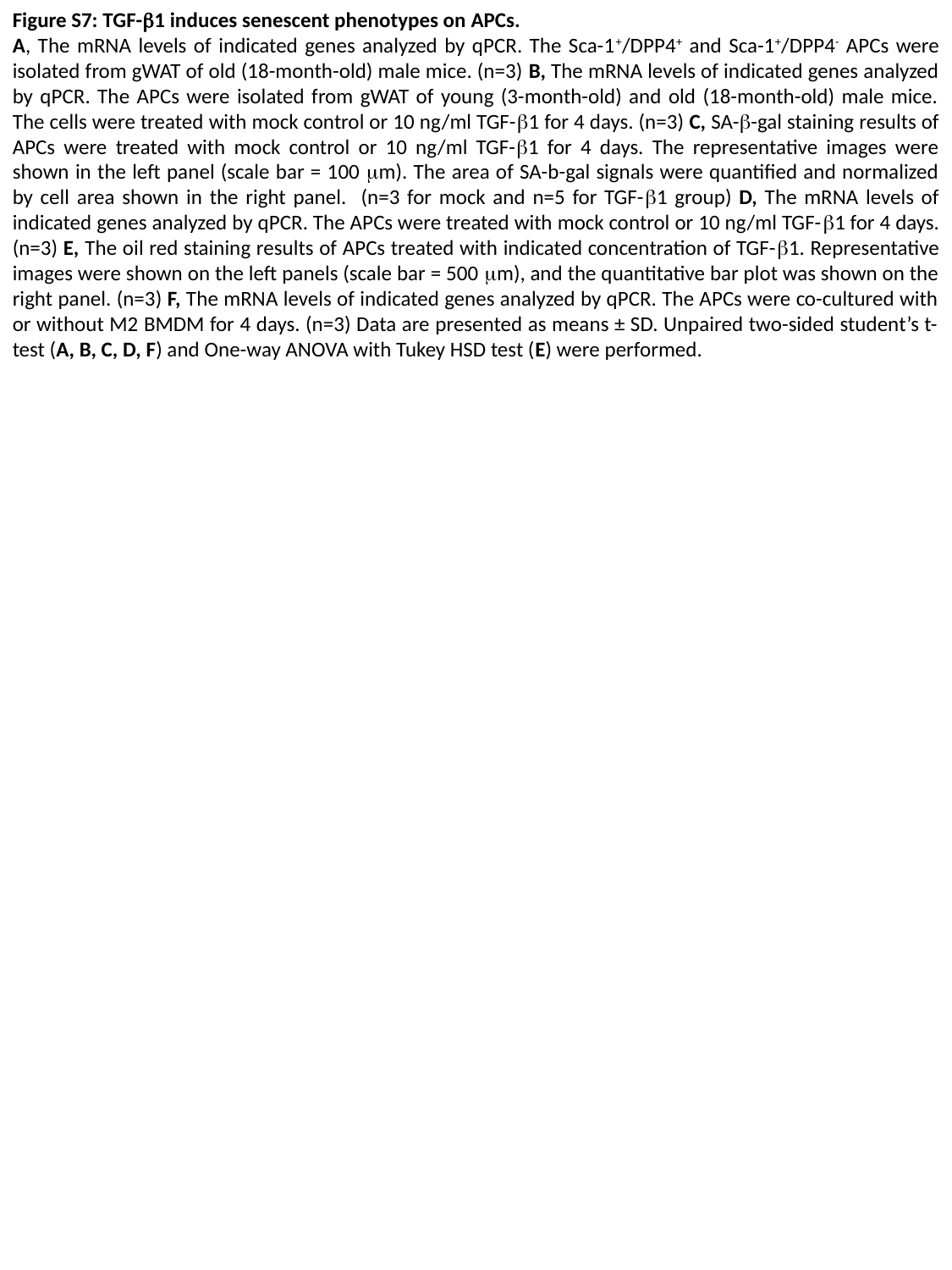

Figure S7: TGF-b1 induces senescent phenotypes on APCs.
A, The mRNA levels of indicated genes analyzed by qPCR. The Sca-1+/DPP4+ and Sca-1+/DPP4- APCs were isolated from gWAT of old (18-month-old) male mice. (n=3) B, The mRNA levels of indicated genes analyzed by qPCR. The APCs were isolated from gWAT of young (3-month-old) and old (18-month-old) male mice. The cells were treated with mock control or 10 ng/ml TGF-b1 for 4 days. (n=3) C, SA-b-gal staining results of APCs were treated with mock control or 10 ng/ml TGF-b1 for 4 days. The representative images were shown in the left panel (scale bar = 100 mm). The area of SA-b-gal signals were quantified and normalized by cell area shown in the right panel. (n=3 for mock and n=5 for TGF-b1 group) D, The mRNA levels of indicated genes analyzed by qPCR. The APCs were treated with mock control or 10 ng/ml TGF-b1 for 4 days. (n=3) E, The oil red staining results of APCs treated with indicated concentration of TGF-b1. Representative images were shown on the left panels (scale bar = 500 mm), and the quantitative bar plot was shown on the right panel. (n=3) F, The mRNA levels of indicated genes analyzed by qPCR. The APCs were co-cultured with or without M2 BMDM for 4 days. (n=3) Data are presented as means ± SD. Unpaired two-sided student’s t-test (A, B, C, D, F) and One-way ANOVA with Tukey HSD test (E) were performed.

## Slide 12
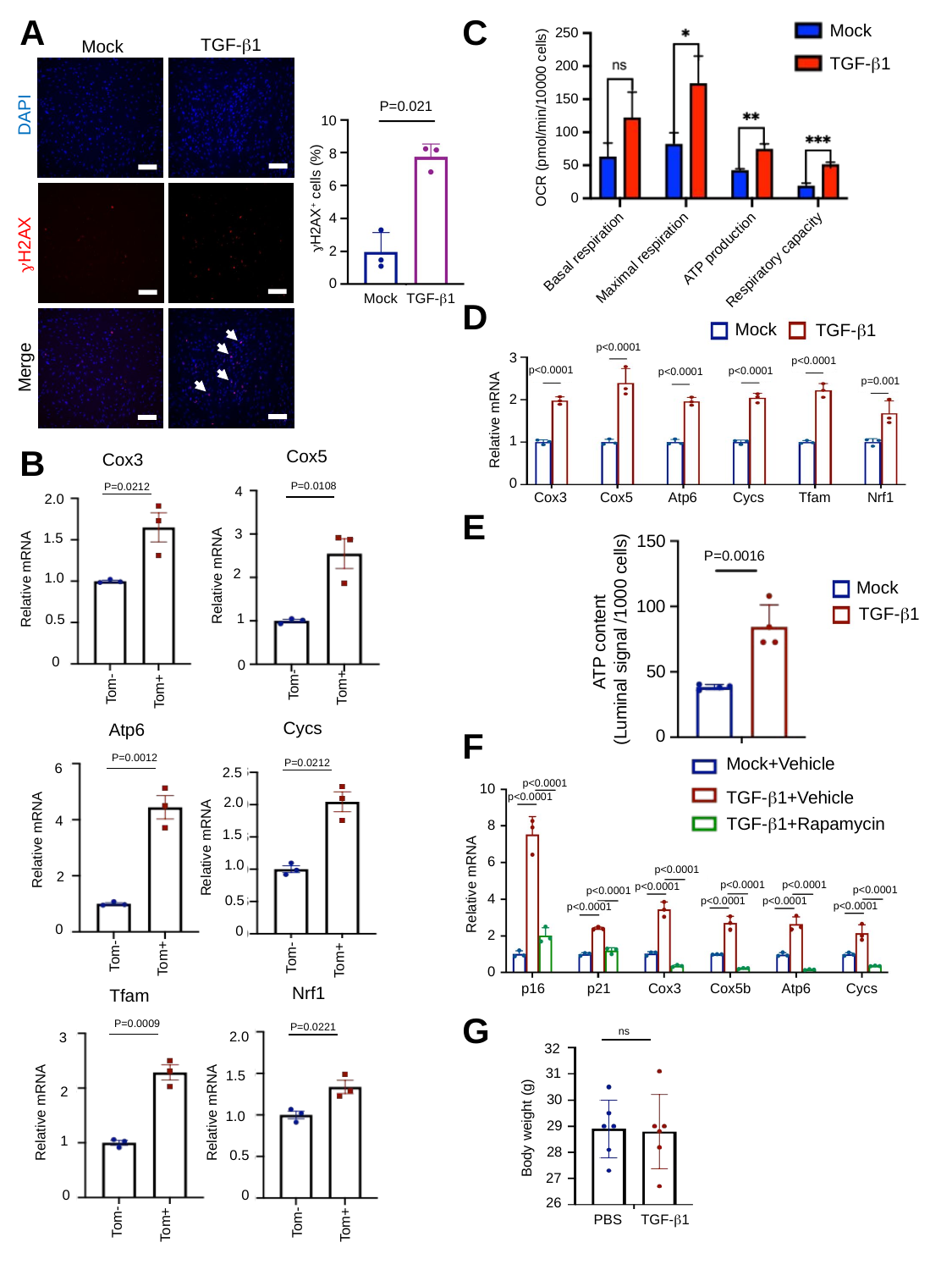

A
C
Mock
250
TGF-b1
200
150
OCR (pmol/min/10000 cells)
100
50
0
ATP production
Respiratory capacity
Basal respiration
Maximal respiration
TGF-b1
Mock
DAPI
gH2AX
Merge
P=0.021
10
8
6
gH2AX+ cells (%)
4
2
0
Mock
TGF-b1
D
Mock
TGF-b1
p<0.0001
3
p<0.0001
p<0.0001
p<0.0001
p<0.0001
p=0.001
2
Relative mRNA
1
0
Cox3
Cox5
Atp6
Cycs
Tfam
Nrf1
B
Cox5
P=0.0108
3
2
Relative mRNA
1
0
Tom+
Tom-
4
Cox3
P=0.0212
1.5
1.0
Relative mRNA
0.5
0
Tom+
Tom-
2.0
E
150
P=0.0016
Mock
100
TGF-b1
ATP content
(Luminal signal /1000 cells)
50
0
Cycs
P=0.0212
1.5
Relative mRNA
1.0
0.5
0
Tom+
Tom-
2.0
2.5
P=0.0012
6
4
Relative mRNA
2
0
Tom+
Tom-
Atp6
F
Mock+Vehicle
p<0.0001
10
TGF-b1+Vehicle
p<0.0001
TGF-b1+Rapamycin
8
6
p<0.0001
Relative mRNA
p<0.0001
p<0.0001
p<0.0001
p<0.0001
p<0.0001
4
p<0.0001
p<0.0001
p<0.0001
p<0.0001
2
0
p16
p21
Cox3
Cox5b
Atp6
Cycs
Nrf1
P=0.0221
1.5
Relative mRNA
1.0
0.5
0
Tom+
Tom-
2.0
Tfam
P=0.0009
3
2
Relative mRNA
1
0
Tom+
Tom-
ns
Body weight (g)
28
26
PBS
TGF-b1
32
31
30
29
27
G

## Slide 13
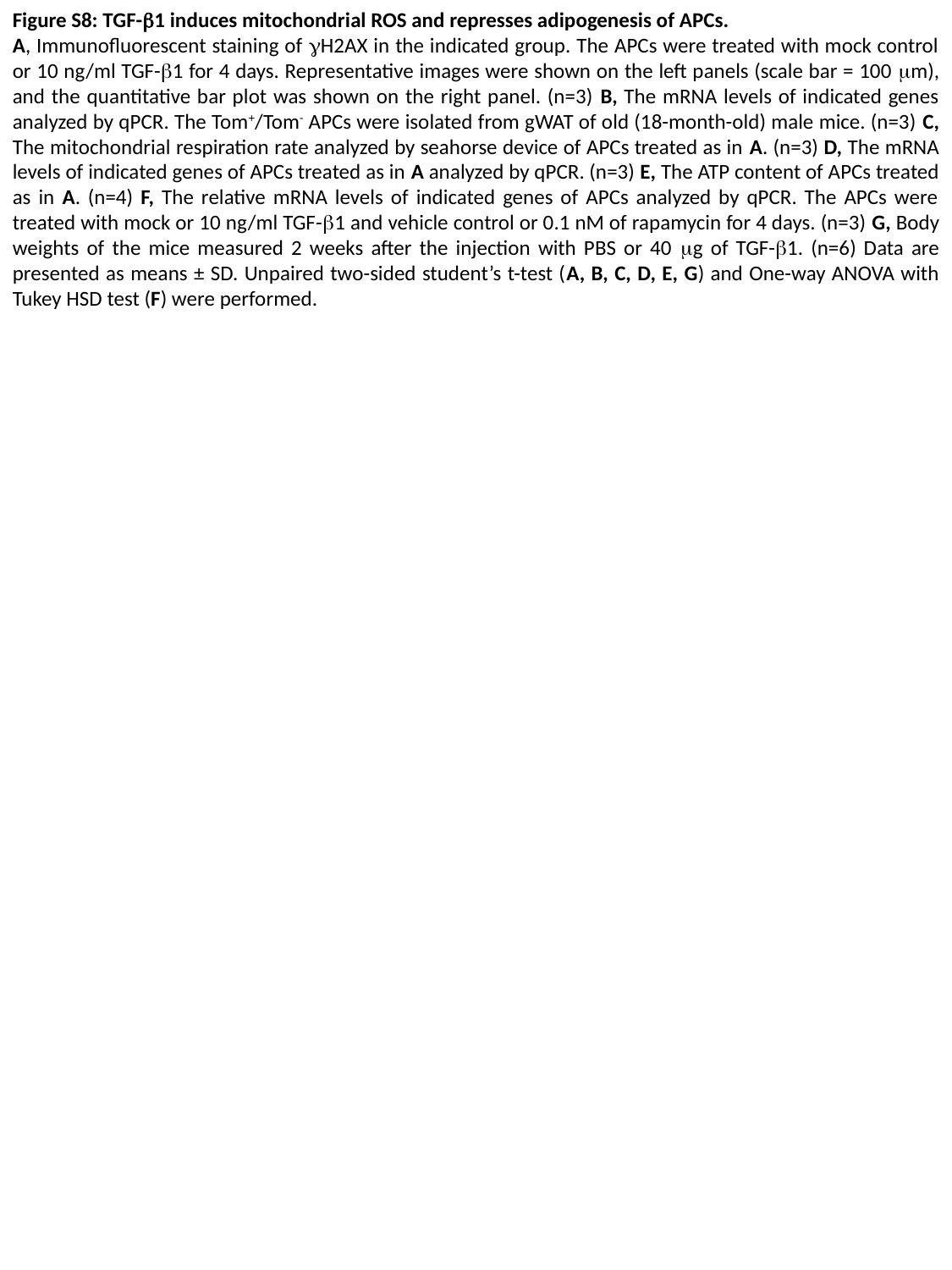

Figure S8: TGF-b1 induces mitochondrial ROS and represses adipogenesis of APCs.
A, Immunofluorescent staining of gH2AX in the indicated group. The APCs were treated with mock control or 10 ng/ml TGF-b1 for 4 days. Representative images were shown on the left panels (scale bar = 100 mm), and the quantitative bar plot was shown on the right panel. (n=3) B, The mRNA levels of indicated genes analyzed by qPCR. The Tom+/Tom- APCs were isolated from gWAT of old (18-month-old) male mice. (n=3) C, The mitochondrial respiration rate analyzed by seahorse device of APCs treated as in A. (n=3) D, The mRNA levels of indicated genes of APCs treated as in A analyzed by qPCR. (n=3) E, The ATP content of APCs treated as in A. (n=4) F, The relative mRNA levels of indicated genes of APCs analyzed by qPCR. The APCs were treated with mock or 10 ng/ml TGF-b1 and vehicle control or 0.1 nM of rapamycin for 4 days. (n=3) G, Body weights of the mice measured 2 weeks after the injection with PBS or 40 mg of TGF-b1. (n=6) Data are presented as means ± SD. Unpaired two-sided student’s t-test (A, B, C, D, E, G) and One-way ANOVA with Tukey HSD test (F) were performed.

## Slide 14
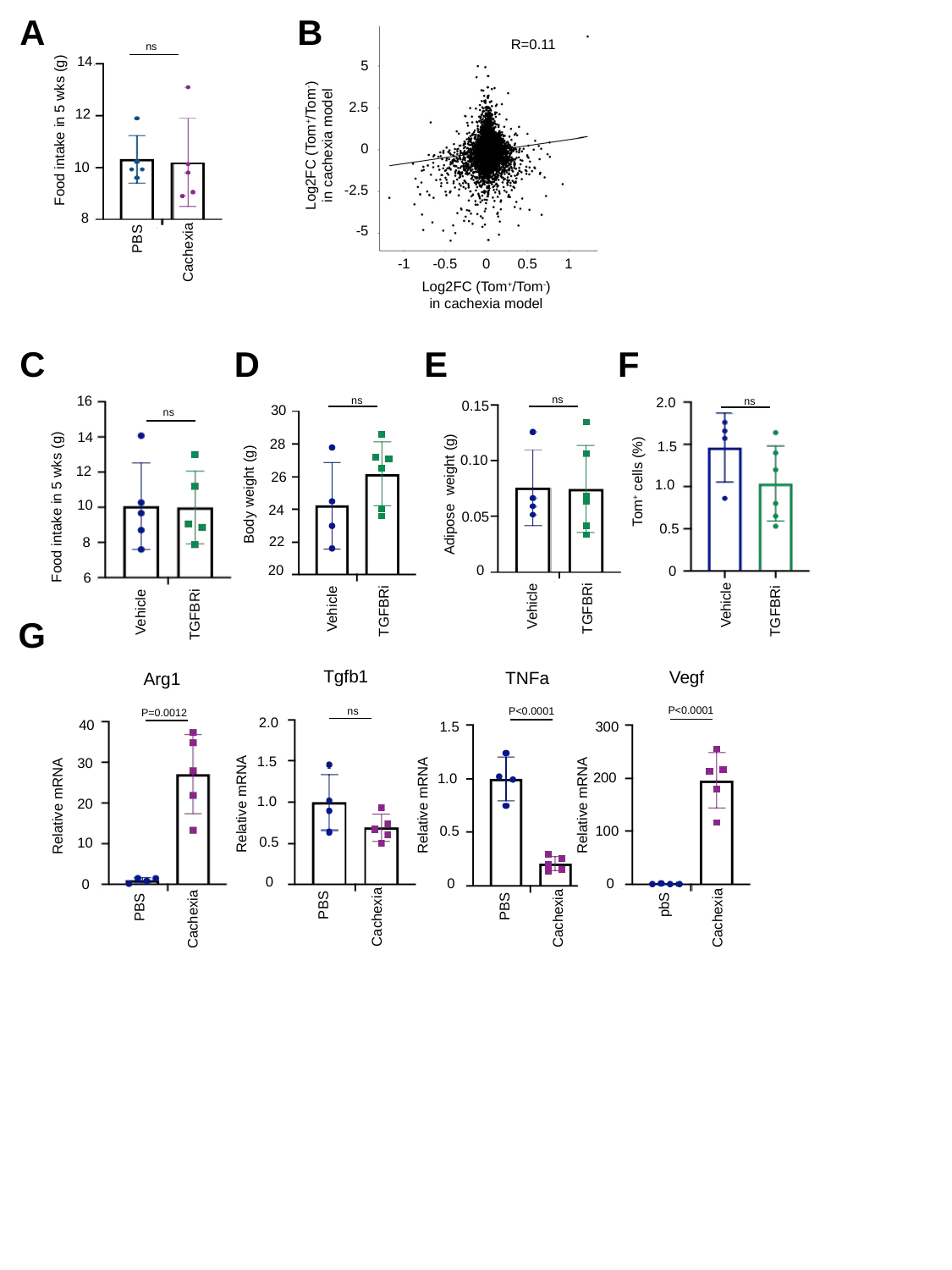

ns
14
12
Food intake in 5 wks (g)
10
8
PBS
Cachexia
A
B
R=0.11
5
2.5
Log2FC (Tom+/Tom-)
in cachexia model
0
-2.5
-5
-1
-0.5
0
0.5
1
Log2FC (Tom+/Tom-)
in cachexia model
C
D
E
F
ns
Tom+ cells (%)
0.5
0
Vehicle
TGFBRi
1.5
1.0
ns
Adipose weight (g)
0.05
0
Vehicle
TGFBRi
0.15
0.10
ns
Body weight (g)
24
20
Vehicle
TGFBRi
30
28
26
22
ns
14
10
Food intake in 5 wks (g)
8
6
Vehicle
TGFBRi
16
2.0
12
G
Tgfb1
ns
2.0
1.5
1.0
Relative mRNA
0.5
0
Cachexia
PBS
Vegf
P<0.0001
300
200
Relative mRNA
100
0
Cachexia
pbS
TNFa
P<0.0001
1.5
1.0
Relative mRNA
0.5
0
Cachexia
PBS
Arg1
P=0.0012
40
30
20
Relative mRNA
10
0
Cachexia
PBS

## Slide 15
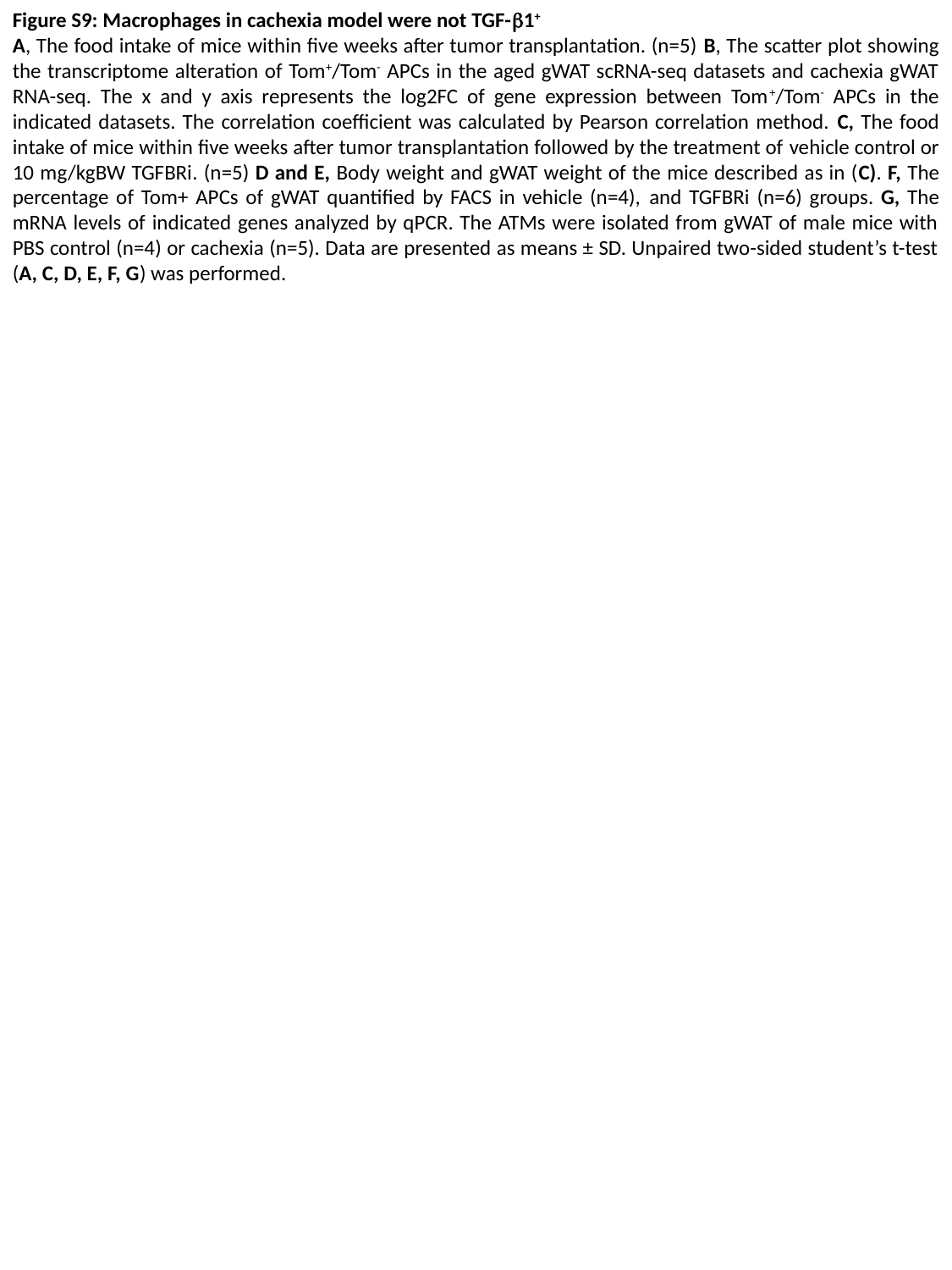

Figure S9: Macrophages in cachexia model were not TGF-b1+
A, The food intake of mice within five weeks after tumor transplantation. (n=5) B, The scatter plot showing the transcriptome alteration of Tom+/Tom- APCs in the aged gWAT scRNA-seq datasets and cachexia gWAT RNA-seq. The x and y axis represents the log2FC of gene expression between Tom+/Tom- APCs in the indicated datasets. The correlation coefficient was calculated by Pearson correlation method. C, The food intake of mice within five weeks after tumor transplantation followed by the treatment of vehicle control or 10 mg/kgBW TGFBRi. (n=5) D and E, Body weight and gWAT weight of the mice described as in (C). F, The percentage of Tom+ APCs of gWAT quantified by FACS in vehicle (n=4), and TGFBRi (n=6) groups. G, The mRNA levels of indicated genes analyzed by qPCR. The ATMs were isolated from gWAT of male mice with PBS control (n=4) or cachexia (n=5). Data are presented as means ± SD. Unpaired two-sided student’s t-test (A, C, D, E, F, G) was performed.
